# Inflammation-induced TRPV4 channels exacerbate blood–brain barrier dysfunction in multiple sclerosis

**DOI:** 10.1186/s12974-024-03069-9

**Published:** 2024-03-23

**Authors:** Cathrin E. Hansen, Alwin Kamermans, Kevin Mol, Kristina Berve, Carla Rodriguez-Mogeda, Wing Ka Fung, Bert van het Hof, Ruud D. Fontijn, Susanne M. A. van der Pol, Laura Michalick, Wolfgang M. Kuebler, Boyd Kenkhuis, Willeke van Roon-Mom, Wolfgang Liedtke, Britta Engelhardt, Gijs Kooij, Maarten E. Witte, Helga E. de Vries

**Affiliations:** 1 Department of Molecular Cell Biology and Immunology, Amsterdam UMC Location Vrije Universiteit Amsterdam, De Boelelaan 1117, Amsterdam, The Netherlands; 2 Amsterdam Neuroscience, Amsterdam UMC, Amsterdam, The Netherlands; 3 MS Center Amsterdam, Amsterdam UMC Location VU Medical Center, Amsterdam, The Netherlands; 4 Department of Biomedical Engineering and Physics, Amsterdam UMC Location University of Amsterdam, Meibergdreef 9, Amsterdam, The Netherlands; 5 Amsterdam Institute for Immunology and Infectious Diseases, Amsterdam UMC, Amsterdam, The Netherlands; 6 Theodor Kocher Institute, University of Bern, Bern, Switzerland; 7 Institute of Physiology, Corporate member of the Freie Universität Berlin and Humboldt Universität to Berlin, Charité-Universitätsmedizin Berlin, Berlin, Germany; 8 DZHK (German Centre for Cardiovascular Research), Partner Site Berlin, Berlin, Germany; 9 Keenan Research Centre for Biomedical Science, St. Michael’s Hospital, Toronto, ON Canada; 10 Departments of Surgery and Physiology, University of Toronto, Toronto, ON Canada; 11 Department of Human Genetics, Leiden University Medical Center Leiden, Leiden, The Netherlands; 12 Department of Neurology, Duke University, Durham, NY USA; 13 Department of Molecular Pathobiology, New York University College of Dentistry, New York, USA; 14 UK Dementia Research Institute at University of Edinburgh, Edinburgh, UK

**Keywords:** Multiple sclerosis, Blood–brain barrier, TRPV4, Vessel-associated microglia, T cells, TNFα

## Abstract

**Background:**

Blood–brain barrier (BBB) dysfunction and immune cell migration into the central nervous system (CNS) are pathogenic drivers of multiple sclerosis (MS). Ways to reinstate BBB function and subsequently limit neuroinflammation present promising strategies to restrict disease progression. However, to date, the molecular players directing BBB impairment in MS remain poorly understood. One suggested candidate to impact BBB function is the transient receptor potential vanilloid-type 4 ion channel (TRPV4), but its specific role in MS pathogenesis remains unclear. Here, we investigated the role of TRPV4 in BBB dysfunction in MS.

**Main text:**

In human post-mortem MS brain tissue, we observed a region-specific increase in endothelial TRPV4 expression around mixed active/inactive lesions, which coincided with perivascular microglia enrichment in the same area. Using in vitro models, we identified that microglia-derived tumor necrosis factor-α (TNFα) induced brain endothelial TRPV4 expression. Also, we found that TRPV4 levels influenced brain endothelial barrier formation via expression of the brain endothelial tight junction molecule claudin-5. In contrast, during an inflammatory insult, TRPV4 promoted a pathological endothelial molecular signature, as evidenced by enhanced expression of inflammatory mediators and cell adhesion molecules. Moreover, TRPV4 activity mediated T cell extravasation across the brain endothelium.

**Conclusion:**

Collectively, our findings suggest a novel role for endothelial TRPV4 in MS, in which enhanced expression contributes to MS pathogenesis by driving BBB dysfunction and immune cell migration.

**Supplementary Information:**

The online version contains supplementary material available at 10.1186/s12974-024-03069-9.

## Introduction

Multiple sclerosis (MS) is a neuro-inflammatory disease of the central nervous system (CNS) with a typical onset in early adulthood [1]. Pathologically, MS is characterized by focal inflammatory and demyelinating lesions, caused by the infiltration of autoreactive immune cells across the brain microvasculature, eventually leading to axonal damage and neurodegeneration [2–4]. In health, the specialized brain microvascular endothelial cells (brain ECs) form the blood–brain barrier (BBB) to protect the CNS from potentially harmful peripheral molecules and ensure regulated influx and efflux of compounds. Next to the specific expression of transporters and efflux pumps, the unique barrier characteristics of the BBB are established through junctional complexes at endothelial cell–cell contact sites [5]. These complexes limit paracellular permeability and regulate molecule passage by tight junction (TJ) proteins like claudin-5 (Cldn5) and adherens junction (AJ) proteins such as VE-cadherin (VE-Cad) [6, 7]. In MS, these protective properties are affected early in the disease course, leading to endothelial inflammation and barrier dysfunction [8–10]. Consequently, the inflamed BBB allows for enhanced peripheral immune cell trafficking into the parenchyma and leakage of systemic components, which activate glial cells and impact neuronal viability [11–15]. Hence, BBB dysfunction and inflammation are driving events in MS lesion formation, which presents a mandate for elucidating the underlying mechanisms of these processes [16, 17].

The pathobiology provoking BBB dysfunction in MS is complex and to date insufficiently understood. However, an increase in intracellular calcium levels in brain ECs seems to play an important role [18–22]. Cytoplasmic calcium levels are normally tightly controlled to secure BBB integrity as they impact the cytoskeleton and TJ dynamics [19]. However in MS, pro-inflammatory mediators, such as cytokines, can induce calcium influx in brain ECs and subsequently activate downstream pathways, thereby disrupting BBB homeostasis [23–25]. Altered intracellular calcium levels in response to inflammation and other pathological stressors, are regulated via ion channels, yet little is known about their expression or mode of action as a contributor to MS [26–29].

Previous reports suggest that members of the transient receptor potential (TRP) ion channel family are essential calcium regulators for brain endothelial function [30–33]. Especially, the polymodal TRP cation channel V4 (TRPV4) regulates BBB integrity, mediates shear stress responses and cell signaling pathways [34–39]. Interestingly, agonist-mediated activation of TRPV4 induces disassembly and degradation of brain endothelial junctional molecules [40, 41]. Moreover, channel activation stimulates the NFκB pathway and promotes the transition of peripheral arterial and venous ECs to a more pro-inflammatory phenotype [42–44]. In the context of MS, BBB dysfunction is orchestrated by various mediators that can also activate TRPV4, which renders endothelial TRPV4 activation a putative candidate mechanism of BBB dysfunction in MS [45–48].

In this study, our findings depict a region-specific TRPV4 increase in the brain endothelium around inflammatory MS lesions and suggest tumor necrosis factor alpha (TNFα) as one inducer for this enhanced expression. Elevated expression of endothelial TRPV4 causes a barrier dysfunction signature during inflammatory conditions, whereas inhibiting TRPV4 activity attenuates T cell migration. With this work, we promote endothelial TRPV4 as a possible contributor to MS disease pathogenesis, with an important impact on BBB integrity, inflammation, and T cell migration processes.

## Materials and methods

### Human tissue samples

Post-mortem snap-frozen and formalin-fixed paraffin-embedded human white matter (WM) tissue blocks were obtained from clinically diagnosed MS patients (n = 11; mean age of death: 61) and non-neurological controls (NNC) (n = 7; mean age of death: 70) and provided by the MS Center Amsterdam and the Netherlands Brain Bank. Patient demographics are listed in Table 1. Tissue blocks were cut into 5 and 10 µm sections and stored at − 80 ºC (frozen) or room temperature (paraffin) until further use. In MS tissue, peri-lesional areas were defined as distal to the lesion and normal myelination, within the same tissue block. In some analyses, cases were eliminated due to insufficient quality in the corresponding tissue sections or unavailability of the required tissue preservation (for details see Table 1). All donors or their next of kin provided fully informed consent for autopsy and use of material for research from the Netherlands Brain Bank under ethical approval by the Medical Ethics Committee of the Free University Medical Center in Amsterdam (2009/148), project number 1127.

**Table 1:** Clinical and demographic data of MS and NNC subjects

Case ID	Age at death	Sex	PMD (h)	Cause of death	Type of MS	Tissue preservation	Lesion type
MS14	65	M	7:15	Terminal liver cirrhosis	unk	FF, FFPE	1 CIA
MS32	54	M	unk	Progressive dyspnea	unk	FF	1 A/I
MS46	51	F	9:10	Euthanasia	SPMS	FF, FFPE	1 A/I 1 CIA
MS47	70	M	9:25	Euthanasia	SPMS	FF, FFPE	2 CIA
MS68	48	F	9:20	Pneumonia	unk	FF	1 A/I
MS71	67	M	7:55	Euthanasia	unk	FFPE	1 A/I
MS80	61	F	6:25	Pneumonia	SPMS	FFPE	1 A/I
MS85	67	F	11:25	Pneumonia	unk	FF, FFPE	2 A/I
MS113	61	F	10:55	Sepsis	PPMS?	FFPE	1 A/I
MS115	56	M	6:15	Assumed pneumonia	PPMS	FF, FFPE	2 A/I
MS116	66	F	9:30	Euthanasia	SPMS	FF, FFPE	1 A/I, 1 CIA
NNC19	68	M	8:40	Euthanasia	n/a	FF, FFPE	n/a
NNC95	71	F	7:50	Lung carcinoma	n/a	FFPE	n/a
NNC782	69	F	13:00	Pulmonary embolism	n/a	FFPE	n/a
NNC797	59	M	8:00	Euthanasia	n/a	FFPE	n/a
NNC880	77	M	11:25	Pneumonia	n/a	FFPE	n/a
NNC989	71	F	7:50	Lung carcinoma	n/a	FF	n/a
NNC999	74	M	10:20	Euthanasia	n/a	FF	n/a

PMD Post−mortem delay, unk unknown, n/a not applicable, PPMS primary progressive MS, SPMS secondary progressive MS, FF freshly frozen, FFPE formalin−fixed paraffin−embedded, CIA chronic inactive, A/I mixed active/inactive

### Human brain endothelial cell culture and treatment

The immortalized human cerebral microvascular endothelial cell line (hCMEC/D3; RRID:CVCL_U985) was a kind gift of Prof. dr. IA Romero (Open University, Milton Keynes, UK) and Prof. dr. PO Coureaud (Université Paris Descartes, France) [49, 50]. Cells were cultured from passages 29 to 35 in endothelial basal medium-2 (EBM-2) supplemented with 2.5% (v/v) heat-inactivated fetal bovine serum (FBS), growth supplement kit (#CC-3156, #CC-4147; Lonza, Basel, Switzerland), and 1% (v/v) penicillin–streptomycin (P/S) (#15,140–122; Gibco, Thermo Fisher Scientific, Waltham, USA). hCMEC/D3 cells were grown on bovine skin collagen I-coated culture flasks (#C5533; Sigma-Aldrich) until confluent unless stated otherwise. Cells were maintained at 37 °C and 5% CO_2_ and routinely screened for the presence of mycoplasma. Before treatment, cells were cultured in starvation medium with 0–0.5% FBS for 16–18 h. Stimulation with cytokines (recombinant human transforming growth factor-β (10 ng/ml, rhTGFβ) (#130-095-067; Miltenyi Biotec, Bergisch Gladbach, Germany), rhTNFα, Interferon gamma (rhIFNγ), (both 5 ng/ml) Interleukin-1 beta (10 ng/ml, rhIL1β) (#300-01A, #300-02-B, #200-01B; all PeproTech)) or channel activity modulation compounds (GSK2193874 (1000 nM), GSK1016790A (100 nM) [51, 52]; both Tocris, Ellisville, USA), occurred in starvation medium. Dimethyl sulfoxide (DMSO) (≤ 0.02%) was used as a vehicle for channel modulation experiments.

### Lentiviral-mediated knock down and overexpression of TRPV4

Lentiviral vectors were used to overexpress or knock down TRPV4 expression in hCMEC/D3 cells and transduction was performed as previously described [53]. In short, sub-confluent HEK293T cells were co-transfected with the target plasmid and helper plasmids for packaging (pMD2G, pRRE, pRSV/pREV) using calcium phosphate. Virus-containing supernatant was collected after 48 h and concentrated using Amicon Ultra15 filters (UFC910024; Merck, Darmstadt, Germany). hCMEC/D3 cells were transduced by adding the concentrated supernatant 4–6 h after seeding and 24 h later selected using puromycin treatment (2 ng/ml, P7255; Sigma Aldrich). TRPV4 knock down, via short hairpin RNA (shTRPV4), was most efficient using the TRCN0000045040 construct, based on reference sequence: NM_021625.5 and coding for 5′-CCAGAACTTGGGCATCATCAA-3′. For expression of human c-terminally EYFP-tagged TRPV4 (NM_021625.5, TRPV4OE) under the control of the PGK promoter, the respective DNA sequences were purchased through BaseGene (Leiden the Netherlands) and cloned in a third-generation lentiviral vector, pLV-CMV-MCS-IRES-SIN. Control cells were transduced with an empty vector (EV) or a non-targeted shRNA (NTC; SHC002, Sigma-Aldrich). Knock down and overexpression efficiency were accessed using quantitative real-time PCR (qRT-PCR) and Western Blot.

### Human iPSC-derived microglia

The generation of human induced pluripotent stem cells (hiPSC) from a NNC was approved by the LUMC scientific ethical committee and informed consent was obtained (NL45478.058.13/P13.080) [54]. The iPSC-derived microglia (hiPSC-MG) were generated following the protocol of Kenkhuis et al. [55]. Briefly, mesodermal embryoid bodies were developed from iPSCs and plated in medium supplemented with macrophage colony-stimulating factor (rhM-CSF, 100 µg/ml; 300–25, Peprotech) and Interleukin 3 (25 µg/ml; #200-03-B, Peprotech). Precursors were differentiated into iPSC-MG for two weeks with Interleukin 34 (20 µg/ml; #200-34, Peprotech) and granulocyte–macrophage (GM)-CSF (2 µg/ml; #300–03, Peprotech), changing the medium every other day (Additional file 1: Fig. S2d). Matured iPSC-MG were treated with Interleukin 4 (IL-4) (20 ng/ml; #11,340,043, ImmunoTools, Friesoythe, Germany) and Interleukin 13 (IL-13) (20 ng/ml; #200–13, Peprotech) for 48 h (anti-inflammatory) and with lipopolysaccharide (LPS) (100 ng/ml; L2630, Sigma Aldrich) and IFNγ (20 ng/ml; #300–02, Peprotech) (pro-inflammatory) for 24 h (day 0). Untreated matured iPSC-MG were used as a control (resting). After treatment, the cells were washed twice to remove LPS and cytokines and the conditioned medium was harvested after 24 h (day 1). Cell phenotypes were confirmed for every batch by qPCR analysis (Additional file 1: Fig. S2e). The medium was stored at − 20 °C until applied on the hCMEC/D3 cells for 24 h. For additional treatment, the conditioned medium was supplemented with a TNF inhibitor (100 ng/ml; Etanercept, Sigma-Aldrich).

### Calcium imaging

Intracellular calcium recordings were performed in serum-free, phenol red-free HBSS (#14,175,129; Gibco) supplemented with 2 mM CaCl_2_ (C7902, Sigma-Aldrich) and 1 mM glucose (#8337, Merck), and quantified by fluorescent lifetime imaging (FLIM). In short, hCMEC/D3 cells were grown in 8-well Ibidi slides (#80,826; ibidi, Munich, Germany) until confluent and loaded with 1–2 µM Oregon Green 488 BAPTA-1 AM (OGB-488) or X-Rhod-1 AM (O6807, X14210, Invitrogen, Thermo Fisher Scientific), in the presence of pluronic and probenecid (F-127, P36400; Thermo Fisher Scientific). After 20 min cell slides were mounted on a 63 × glycerol or oil objective of a Leica TCS-SP8 FALCON microscope (NA 1.3, 1.4; Leica, Wetzlar, Germany). Recordings were made at 37 °C in 5% CO_2_ and 80% humidity. Fluorophores were excited with a pulsed diode laser (PicoQuant) and photon arrival times were recorded with two HyD detectors adjusted to count photons at approximately equal rates using spectral ranges of 516–542 nm; 543–629 nm (OGB-488) and 621–637 nm; 637–672 nm (X-Rhod-1) respectively. Autofocus control was used to prevent focus drift when stimuli were applied. Excitation and emission wavelength were: OGB-488 λ_ex_: 480 nm, λ_em_: > 502 nm; X-Rhod-1 λ_ex_: 580 nm, λ_em_: > 620 nm. For analysis, data was fitted in a double-exponential manner and mapped to a weighted mean lifetime scale of τ_1_: 0.17 ns, τ_2_: 2.91 ns (OGB-488); τ_1_: 0.55 ns, τ_2_: 4.01 ns (X-Rhod-1).

### Electric cell-substrate Impedance sensing (ECIS)

The transendothelial electric resistance (TEER) of hCMEC/D3 cells was assessed with the ECIS™ Model 1600R (Applied BioPhysics, Troy, NY) as previously reported [56, 57]. In short, cells were seeded at a density of 100,000 cells into 8W10 + ECIS arrays (#72,040, Ibidi). Impedance Z was measured at multiple frequencies over a time course of 60–170 h. To quantify the resistance [ohm], the data at 4000 Hz was normalized to the resistance at the time of cell attachment or treatment start.

### Human T cell isolation and transmigration assay

Human T cells were isolated from buffy coats (Sanquin Blood Bank, Amsterdam, The Netherlands) using a Ficoll density gradient (F4375; Sigma-Aldrich) and negative selection kits (#130–096-533, # 130–096-495; Miltenyi Biotec, Bergisch Gladbach, Germany) [58]. Purity was tested using flow cytometry and cells were stored in liquid nitrogen until use. For transmigration assays, T cells were cultured in Roswell Park Memorial Institute (RPMI) 1640 medium (#11875093; Gibco) supplemented with 10% FBS and 1% (v/v) P/S (#15140–122, Gibco) (T cell medium). T cells were stimulated using soluble 1 µg/mL αCD3 and 1 µg/mL αCD28 antibodies (#317326, # 302934; BioLegend) and 10 ng/ml rhIL-2 (#200–02; PeproTech, Rocky Hill, USA) for 2 h (CD4^+^) or 18 h (CD8^+^) respectively. hCMEC/D3 cells were grown to confluency on 6,5 mm transwell filters (5 µm pore size, #3421; Corning, Glendale, USA) and partially stimulated with TNFα/IFNγ (5 ng/ml) in serum-free, growth-factor free medium (#11111044, Gibco) for 16–18 h. T cells were added in quadruplicates at a density of 1 × 10^6^/well in T cell medium for 4 h. Endothelial cell-free wells with the same T cell number served as total cell input control. Migrated (lower well) and non-migrated (upper well) cells were counted manually under the microscope using a Neubauer chamber and further examined by flow cytometry.

### Flow cytometry

Migrated and non-migrated human T cells were stained with fixable viability dye (FVD) eFluor780 (#65–0865-14, eBioscience, Thermo Fisher), CD4-PE (#300508, BioLegend), CD8-PE (#21270084, ImmunoTools), CD49d-APC (#559881) and CD11a-BV650 (#745344) (all BD Biosciences, Franklin Lakes, USA). Surface expression was calculated as median fluorescence intensity (MFI). Using a classical gating strategy, cells were selected by using FSC-A and SCC-A and singlets based on the FSC-A vs. FSC-H. Dead cells were excluded by FVD eFluor780 positivity and the gate defined on the positive CD4/CD8 population (Additional file 1: Fig. S4 a, c). Measurements were performed with the BD LSRFortessa™ X-20 (BD Biosciences) and analyzed with Flow Jo v10.7.

### Immunostainings

Sections were defrosted at room temperature (20 min) and subjected to acetone fixation (10 min, frozen) or deparaffinated followed by epitope retrieval at 95 ºC for 30 min. Tissues were incubated for 30 min with a blocking solution containing 0.01% Triton X-100 (Sigma-Aldrich) and corresponding species normal serum or BSA 10% in PBS. The primary antibody cocktail was applied overnight at 4 ºC (Table 2) followed by 60 min incubation at room temperature with appropriate secondary antibodies conjugated to Alexa dyes (Alexa488, 555, 647, 750; Thermo Fisher Scientific). All immunofluorescent-stained sections were counterstained for DNA using Hoechst (1:1000, Molecular Probes, Eugene, USA). After washing, sections were embedded in Mowiol (in-house) and stored at 4 ºC until image acquisition. For chromogenic immunohistochemistry, primary antibodies were detected using the EnVision + visualization system with 3,3′-diaminobenzidine (K500711-2; DAKO, Aligent, Santa Clara, USA). Sections were subsequently counterstained with hematoxylin, dehydrated, and embedded in Entellan medium (#13073–00, Merck). For immunocytochemistry, cells were fixed with 4% PFA, absolute (> 99%) ethanol, or methanol for 10 min at RT, permeabilized for 5 min with 0.05% Triton X-100 in PBS and blocked with 10% normal serum or BSA in PBS. The cell staining procedure was equivalent to tissue sections.

**Table 2:** Primary antibody details

Target	Host	Dilution	Retrieval/fixation	Supplier	Cat.#	Use
CD3 (F7.2.38)	Mouse	1:50	None	Dako	M7254	human T cells (ICC)
CD206	Mouse	1:50	Acetone	Biolegend	321,116	IHC FF
CLDN5	Rabbit	1:200	None	Invitrogen	#34–1600	hCMEC/D3
Collagen IV	Rabbit	1:200	Acetone	Abcam	ab6581	IHC FF
GAPDH	Goat	1:500	None	Santa Cruz	SC-20357	WB hCMEC/D3
HLA-DR (LN3)	Mouse	1:500	Citrate, Tris, Acetone	In house		IHC FFPE, FF
IBA1	Goat	1:500	Citrate,Tris	Abcam	AB5076	IHC FFPE
P2RY12	Rabbit	1:100	Citrate,Tris	Atlas antibodies	HPA014518	IHC FFPE
P65	Rabbit	1:500	PFA (4%)	Abcam	ab16502	ICC hCMEC/D3
Phalloidin		1:100–250	None	Molecular Probes	A12379, R415	ICC hCMEC/D3
TNFα	Rabbit	1:200	Acetone	Origene	PP1071P1	IHC FF
TRPV4 intrac	Rabbit	1:50	Acetone	Alomone Labs	ACC034	IHC FF,WB
TRPV4 extrac	Rabbit	1:50	Acetone	Alomone Labs	ACC124	IHC FF
TRPV4	Rabbit	1:100	Acetone	Invitrogen	# PA5-41066	IHC FF
UEA-1		1:1000	Acetone	Vector labs	B-1065	IHC FF

Citrate pH 6.0; Tris 10 mM Tris / 1 mM EDTA, pH 9.0

### Microscopy and image acquisition

Confocal images were acquired using a Leica SP8 confocal microscope (Leica, Wetzlar, Germany) with a 63 × oil immersion objective with a 1.40 numerical aperture or the Olympus IX81-ZDC microscope with the RCM1 super-resolution imaging extension (Confocal.nl, Amsterdam, The Netherlands; 60 × oil). TRPV4 mean intensity was measured within an endothelial mask based on Ulex Europaeus Agglutinin I (UEA-I) staining and stacks of images of 27/28 z-series with a z-step of 0.15 μm were taken. For the analysis of vessel-associated microglia and TNFα reactivity, the tissue slides were assessed with a 20 × overview scan and imaged at 60x (Olympus X line, 1.42 NA, oil) using the Olympus VS200 slide scanner. We evaluated the microglia volume close to the vasculature based on purinergic receptor P2RY12, as a microglia marker, and UEA-I. Using a 3D analysis, we created a 5 µm circumference around the vasculature and assessed the proportion of P2RY12 signal contained within this region. Images were deconvolved using Huygens Professional 21.10 software (scientific volume imaging B.V.) and (if possible) batch-analyzed using NIS elements (version 5.30.03, Nikon Europe B.V., Amsterdam, The Netherlands). The quality of the images and batch analysis were inspected by comparing image histograms, background mean intensity, and image quality.

### Western Blot

Western Blots were performed similarly to previous work [59]. In short, hCMEC/D3 cells were lysed on ice in Laemmli Buffer and snap-frozen (2×, #161–0737; Bio-rad, Hercules, USA). Protein extracts were centrifuged and heated to 95 °C for 5 min before applying them on the SDS-PAGE (10–15%). After transfer to a PVDF membrane, blots were blocked in 5% milk-TBST for 1 h at room temperature and incubated with primary antibodies in a blocking solution (Table 2) at 4 °C overnight. IRDye secondary antibodies (LI-COR) were incubated for 1 h at room temperature and visualized by Azure Sapphire Biomolecular Imager. GAPDH or β-actin were used as loading control for normalization and densitometric analysis was performed with ImageJ (version 1.49v). Raw blots are added in Additional file 2: Fig. S1a–c.

### RNA isolation and real-time quantitative polymerase chain reaction (qRT-PCR)

Total RNA was extracted from cells using TRIzol (#15596–018, Thermo Fisher Scientific) and the quantity was assessed with a Nanophotometer (Implen, Westlake Village, USA). The High-Capacity cDNA Reverse Transcription Kit (#4368813, Thermo Fisher Scientific) was used to synthesize cDNA and transcripts of interest were detected with SYBR Green (#4,309,155, Thermo Fisher Scientific) using either the Applied Biosystems Viia7 real-time PCR machine or QuantStudio™ 3 Real-Time PCR System (#4453543, #A28567, Thermo Fisher Scientific). Expression was measured in biological and technical triplicates, normalized to housekeeping genes *GAPDH* (brain ECs) and *POL2RF* (hiPSC MG) using the 2^−ΔΔ^CT relative quantification method. Primer sequences are summarized in Table 3.

**Table 3:** Primer details

Target	Forward primer (5′-3′)	Reverse primer (5′-3′)
ALCAM	ACA CGA TGA GGC AGA CGA GAT	CCC ACA ATT AGT TTT GCC TGG
ANG1	CAG TGG CTG CAA AAA CTT GAG A	AGT CTG AGA GAG GAG GCT GG
CAV1	CTC AAC TCG CAT CTC AAG CTG G	TGT CAA AGG AGT GCG TAG TCA
CCL2	CAG CCA CCT TCA TTC CCC	TGC ACT GAG AT
CCL5	GAC ACC ACA CCC TGC TGC T	TAC TCC TTG ATG TGG GCA CG
CLDN5	ACA TTG TCG TCC GCG AGT TT	ACT TCT GCG ACA CGG GCA
COX2	CCT CAG ACA GCA AAG CCT AC	ACA CCT CGG TTT TGA CAT GG
CSF1R	GCTGCCTTACAACGAGAAGTGG	CATCCTCCTTGCCCAGACCAAA
SELE	AAG TTC GCC TGT CCT GAA G	CAG AAA GTC CAG CTA CCA AGG
FN	ACT GTA CAT GCT TCG GTC AG	AGT CTC TGA ATC CTG GCA TTG
FSP1	CTG CAT CGC CAT GAT GTG TA	CCC AAC CAC ATC AGA GGA GT
GAPDH	CCA TGT TCG TCA TGG GTG TG	GGT GCT AAG CAG TTG GTG GTG
GLUT1	CCA TGT GCT TCG GTT TTG TG	AAT CAG GAA GAG AAT ACC CAC G
HLA-DRA	AGCTGTGGACAAAGCCAACCTG	CTCTCAGTTCCACAGGGCTGTT
ICAM1	TAG CAG CCG CAG TCA TAA TGG G	AGG CGT GGC TTG TGT GTT CG
IL1B	CCA AAC CTC TTC GAG GCA CAA	TAC TTC TGC CAT GGC TGC TTC A
Il1R1	CCT GCT ATG ATT TTC TCC CAA TAA A	AAC ACA AAA ATA TCA CAG TCA GAG GTA GAC
INFγ	GAGTGTGGAGACCATCAAGGAAG	TGCTTTGCGTTGGACATTCAAGTC
MFSD2A	TCA TCC TGT TTG TGG GCC	ATG AGG AAG TAG GCA ATG ACG
MERTK	CAGGAAGATGGGACCTCTCTGA	GGCTGAAGTCTTTCATGCACGC
NFκB p50	GCA GCA CTA CTT CTT GAC CAC C	TCT GCT CCT GAG CAT TGA CGT C
P2RY12	ACC AGA GAC TAC AAA ATC ACC C	AGA AAA TCC TCA TCG CCA GG
PECAM1	CTG ATG CCG TGG AAA GCA G	GCA TCT GGC TTG CTG TCT AA
PGP	GTC CCA GGA GCC CAT CCT	CCC GGC TGT TGT CTC CAT A
POLR2F	GAA CTC AAG GCC CGA AAG	TGA TGA TGA GCT CGT CCA C
TGFβ	CTT TCC TGC TTG TCA TGG CC	CCG TGG AGC TGA AGC AAT AG
TMEM119	AGC ACG GAC TCT CTC TTC CAG	GTG CCC CCA GGA CCA GTT C
TNFα	AAA CAA CCC TCA GAC GCC ACA T	AGT GCT CAT GGT GTC CTT TCC A
TREM2	ATGATGCGGGTCTCTACCAGTG	GCATCCTCGAAGCTCTCAGACT
TRPV4	CAC CTG TCC CGC AAG TTC AA	CAT CTC GTG GCG GTT CTC AA
VE-Cad	AAA CAC CTC ACT TCC CCA TC	ACC TTG CCC ACA TAT TCT CC
VIM	CGT GAA TAC CAA GAC CTG CTC	GGA AAA GTT TGG AAG AGG CAG

### Statistical analysis

All analyses were performed blinded and data are shown as the mean ± standard error (SEM) of a minimum of three independent experiments. Statistical tests were performed using GraphPad Prism v9 (GraphPad Software, La Jolla, USA). We used the Shapiro–Wilk test as a test for data normality. For comparing more than two groups, we used a two-tailed one-way analysis of variance (ANOVA) with the Bonferroni test for multiple comparisons or Dunnett’s multiple comparisons test. Non-parametric data of more than two groups was analyzed by Kruskal–Wallis with Dunn’s test. For comparing two experimental groups, a two-tailed Student’s t-test was used. Paired data analysis is indicated by connecting lines. Test details are indicated in the corresponding figure legend. Data were considered statistically significant when p < 0.05 and additional p-values are indicated in the figure legends. For the gene expression heat map, we used the web-based tool MetaboAnalyst (http://www.metaboanalyst.ca, accessed: 01/03/2023) and for the RNA expression data on TRPV4 we used the single nuclei RNA sequencing database of Yang et al., 2022 (https://twc-stanford.shinyapps.io/human_bbb/, accessed: 08/11/2023) [60].

## Results

### Enhanced endothelial TRPV4 expression in peri-lesional MS tissue

**Fig. 1:**
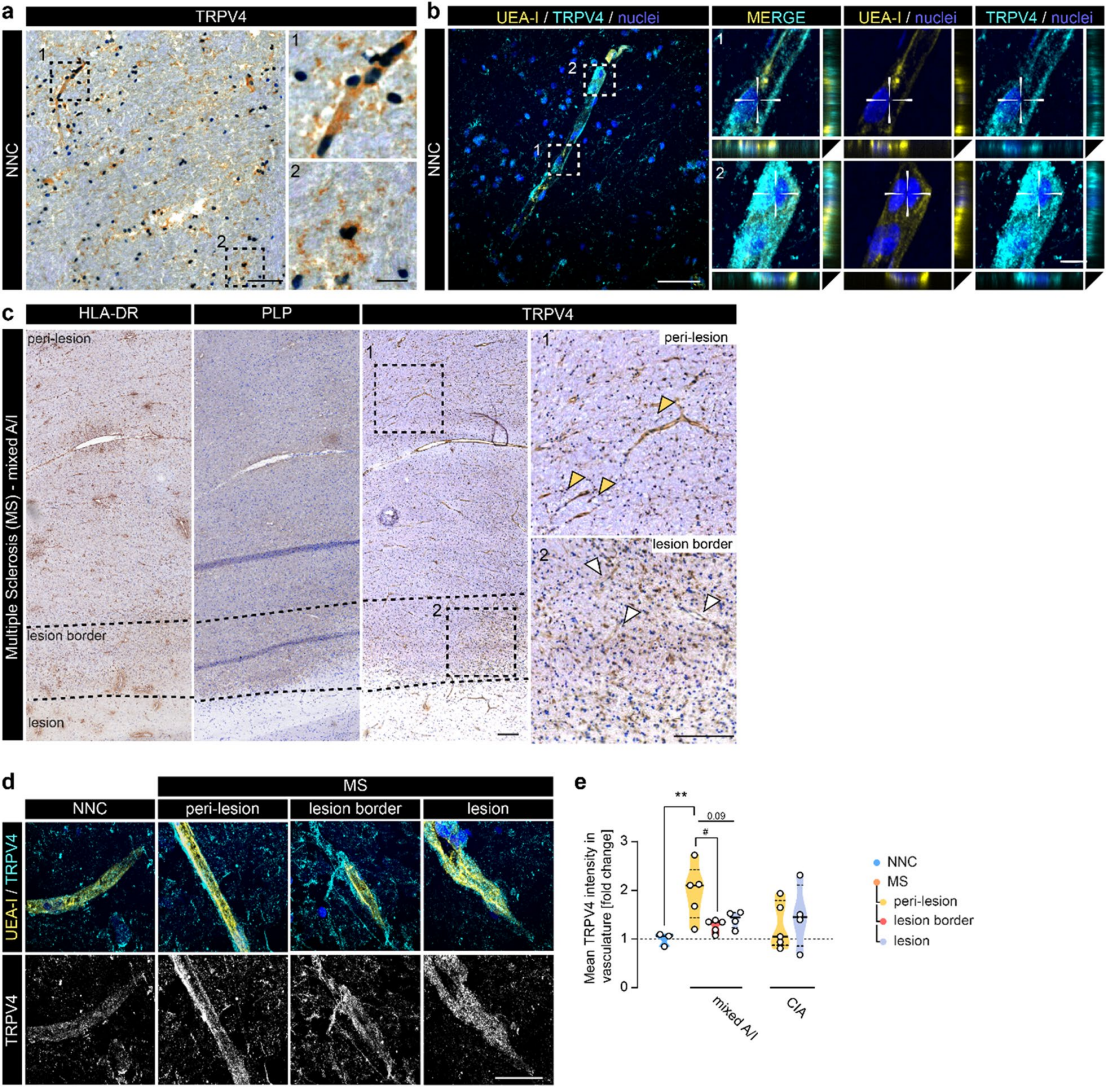
Vascular TRPV4 expression is increased in areas around inflammatory MS lesions. **a** Representative image of TRPV4 immunoreactivity in NNC WM tissue; scale bar: 100 µm. Panels highlight morphologically different types of TRPV4^+^ cells including glial and vascular cells; scale bar: 25 µm. **b** Representative confocal image of UEA-I (endothelial marker) and TRPV4 immunoreactivity in NNC; scale bar: 50 µm. Panels demonstrate TRPV4-UEA-I co-localization; scale bar: 5 µm. **c** Representative images of HLA-DR, PLP, and TRPV4 immunoreactivity in mixed active/inactive (A/I) WM lesion tissue. Overview TRPV4 image shows the staining pattern at low magnification of peri-lesional, lesion border, and lesion tissue respectively (dotted line indicates areas, squares indicate location of panels); scale bar: 200 µm. Panels demonstrate TRPV4 staining in peri-lesional (orange arrowheads) and lesion border tissue (white arrowheads). **d** Representative confocal images of TRPV4-UEA-I immunoreactivity in NNC and MS cases; scale bar: 50 µm. **e** Quantification of TRPV4 mean fluorescent intensity measured within the UEA-I signal (endothelium) in NNC (N cases = 3) and MS (mixed A/I): N cases = 3, N lesions = 5; chronic inactive (CIA): N cases = 4, N lesions = 5). Statistical analysis was performed using one-way ANOVA with Dunnett’s multiple comparisons test, followed by paired one-way ANOVA analysis within the MS cases (#). Violin plots show median ± quartiles (*p < 0.05, **p < 0.01; ^#^p < 0.05)

To investigate the potential role of TRPV4 in the brain endothelium in MS, we first assessed TRPV4 in post-mortem WM brain tissue from NNC. Immunohistochemical evaluation showed prominent TRPV4 reactivity in the vasculature, albeit not exclusively (Fig. 1a). Analysis of a single nuclei RNA sequencing data set confirms ubiquitous expression of TRPV4 in the human brain with the highest counts in the brain endothelium (Additional file 1: Fig. S1a, [60]). Immunofluorescent co-labeling with UEA-I (lectin), used as an endothelial marker, confirmed endothelial TRPV4 immunostaining (Fig. 1b). Next, we visualized TRPV4 reactivity in MS brain tissue. The lesion and lesion border were distinguished by differences in PLP and HLA-DR reactivity from peri-lesional areas, which refers to regions within the same tissue block located distal to the lesion. We observed an intensified vascular TRPV4 immunostaining in the peri-lesional area (orange arrows) compared to the lesion border (white arrows, Fig. 1c). To quantify potential changes in TRPV4 reactivity within MS tissue areas and compared to control, we measured the mean fluorescent intensity of TRPV4 in the endothelium of WM tissue of NNCs and MS patients (Fig. 1e). Within the MS cases, we further categorized the lesions as mixed active/inactive (A/I) and chronic inactive (CIA) based on previous classifications (Fig. 1c, Additional file 1: Fig. S1b, [3]). Strikingly, endothelial immunostaining of TRPV4 was predominantly increased in the peri-lesional area of mixed A/I lesions but not in the more inflamed lesion border or in the lesion itself, while the levels in and around CIA lesions largely varied and did not significantly differ from NNC (Fig. 1d, e). Together, we found a region-specific increase of endothelial TRPV4 in peri-lesional WM around mixed A/I lesions in MS brain tissue.

### Microglia-derived TNFα triggers endothelial TRPV4 expression

**Fig. 2:**
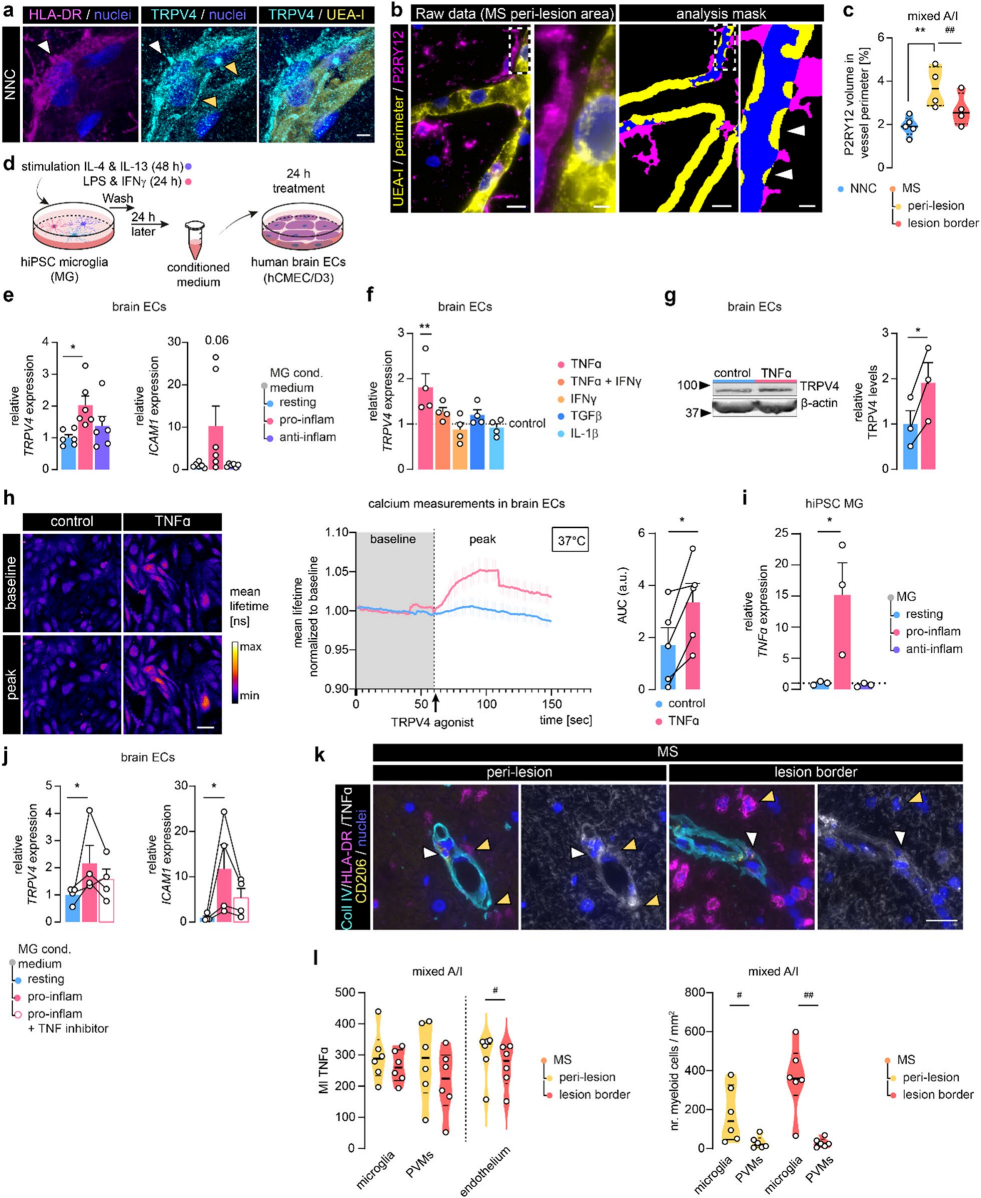
Microglia-derived TNFα induces TRPV4 expression in brain endothelial cells. **a** Representative confocal image of UEA-I, HLA-DR, and TRPV4 immunoreactivity in NNC tissue. The white arrowhead demonstrates TRPV4 in HLA-DR^+^ cells; orange arrowheads highlight endothelial, junctional TRPV4 reactivity; scale bar: 5 µm. **b** Representative images of UEA-I and P2RY12 (microglia) immunoreactivity in MS tissue (left); masks for 3D microglia-vasculature proximity analysis (right). Arrowheads indicate microglia volume (blue) within the vascular perimeter (yellow: 5 µm); scale bar: 10 µm, panel: 2 µm. **c** Quantification of microglia volume within vessel perimeter, N cases = 4–5. **d** Schematic of human brain ECs (hCMEC/D3) treatment with hiPSC microglia conditioned medium (MG cond. medium). **e** *TRPV4* and *ICAM1* measured in brain ECs treated with MG cond. medium, N = 6. **f** *TRPV4* in brain ECs treated with cytokines relative to control, N = 4. **g** Protein levels of TRPV4 measured in TNFα-treated brain ECs normalized to a reference protein, N = 3. **h** Quantification of TRPV4 agonist-mediated calcium response in brain ECs treated with TNFα by the area under the curve (AUC) (GSK1016790A, 100 nM). Measurements were performed at 37 °C and normalized to baseline, N experiments = 5, N cells = 30–40; scale bar: 50 µm. **i** *TNFα* measured in hiPSC MG, N = 3. **j** *TRPV4* and *ICAM1* measured in brain ECs treated with pro-inflammatory cond. MG medium with/without TNF inhibitor, N = 4 **k** Representative images of TNFα, HLA-DR, CD206, and Coll IV immunoreactivity in MS tissue; white arrowheads indicate CD206^+^ perivascular macrophages (PVMs), orange arrowheads indicate microglia (HLA-DR^+^,CD206^−^, outside Coll IV); scale bar: 25 µm. **l** Quantification of TNFα mean intensity in microglia, PVMs, and endothelium and myeloid cell count. Data is shown as the mean ± SEM and statistics were calculated for three groups by one-way ANOVA with Bonferroni or Dunnett's multiple comparisons test or non-parametric Kruskal–Wallis with Dunns test. Comparison of two groups was performed using paired Student’s t-test indicated by connecting lines or within MS tissues (*p < 0.05, **p < 0.01; #p < 0.05, ##p < 0.01)

Higher resolution analysis revealed that brain endothelial cells (ECs) in NNC show both cytoplasmic and junctional localization of TRPV4 (Fig. 2a, orange arrows), suggesting a role for TRPV4 in barrier formation. We observed a higher endothelial TRPV4 signal when HLA-DR^+^ cells were proximate to the vasculature (Fig. 2a, white arrows). HLA-DR^+^ cells themselves showed also TRPV4 reactivity, but its levels did not differ between NNC and MS tissue (Additional file 1: Fig. S2.a, b) [42, 61]. Since in MS, increased numbers of (HLA-DR-expressing) microglia are detected (Additional file 1: Fig. S2c [62]), we hypothesized that the proximity of microglia to the endothelium can play a role in the regulation of endothelial TRPV4 expression. We found an increased volume of microglia, marked by P2RY12, in the direct vicinity of the brain endothelium particularly in peri-lesional areas of MS cases compared to the control (Fig. 2b, c). Moreover, within the MS tissue, microglia volume in proximity to the vasculature was significantly lower in the lesion border compared to peri-lesional areas in mixed A/I lesions (Fig. 2c). Together, these data indicate that endothelial-microglia proximity and/or their interaction is increased in MS peri-lesional tissue, therefore, these microglia are appropriately positioned to mediate the observed endothelial TRPV4 upregulation in peri-lesional white matter.

Endothelial-microglia proximity allows microglia-derived factors to regulate endothelial function [63, 64]. To explore the effects of microglia-secreted factors on brain ECs in vitro we collected conditioned medium of human iPSC-derived microglia (hiPSC MG) (Fig. 2d, Additional file 1: Fig. S2d). hiPSC MG were stimulated with pro- and anti-inflammatory mediators (LPS, INFγ/IL-4 and IL-13, respectively) to model a pro-inflammatory/anti-inflammatory milieu (Additional file 1: Fig. S2d, e) [55, 65]. Importantly, stimulated cells were washed to remove stimuli before the conditioned medium was collected after 24 h. Human brain ECs (hCMEC/D3 cell line [49]) exposed to the conditioned medium of pro-inflammatory hiPSC MG showed increased *TRPV4* expression, concomitant with elevated expression of the intercellular adhesion molecule-1 (*ICAM1*) (Fig. 2e). To better understand which factor(s) within the conditioned medium might evoke endothelial TRPV4 expression, we applied candidate cytokines (TNFα, IFNγ, TGFβ, Il1β) for 24 h to brain ECs separately (Fig. 2f). Solely, TNFα increased TRPV4 expression at both the RNA and protein level, while this effect was not observed with the concurrent addition of IFNγ (Fig. 2f, g, Additional file 1: Fig. S2f). To assess the functional consequence of TNF-induced TRPV4 expression, we next stimulated control and TNFα-treated brain ECs with a selective TRPV4 agonist (GSK1016790A, 100 nM) and measured the intracellular calcium response. TNFα-treated brain ECs displayed a higher calcium response upon TRPV4 activation compared to control cells (Fig. 2h). Corroborating our results, we observed that TNFα was produced and secreted by pro-inflammatory hiPSC MG (Fig. 2i, Additional file 1: Fig. S2g). To confirm that specifically TNFα in the microglia-conditioned medium induces endothelial TRPV4 expression, we simultaneously added a specific TNF inhibitor (Etanercept, 100 ng/ml). Indeed. Etanercept treatment partly reversed the increase in *ICAM1* and *TRPV4* expression in brain ECs resulting in expression levels not significantly different from the control condition (Fig. 2j).

Next to microglia, perivascular macrophages (PVM) are known to contribute to TNFα production in the inflamed CNS [66]. To confirm our hypothesis that endothelial TRPV4 expression in peri-lesional MS tissue is induced by microglia-derived TNFα rather than by PVM-derived TNFα, we visualized TNFα levels together with HLA-DR, CD206 (PVM marker) and collagen IV (Coll IV), a component of the basement membrane, in MS tissues (Fig. 2k). We observed immunoreactivity of TNFα in microglia (HLA-DR^+^, CD206^−^ cells, outside the Coll IV mask) and PVMs (CD206^+^ cells within Coll IV mask) in peri-lesional and lesion border areas (Fig. 2l). TNFα mean intensity was similar for both myeloid cell populations and in both tissue areas. As expected the number of microglia was significantly higher than the number of PVMs (Fig. 2l). TNFα reactivity in the vasculature was significantly lower in the lesion border compared to the peri-lesional tissue. These findings suggest that in MS, brain endothelial TRPV4 expression can be initiated via the secretion of TNFα predominantly by activated microglia as well as PVMs and vasculature.

### TRPV4 levels steer barrier formation and Cldn5 expression at the homeostatic BBB

**Fig. 3:**
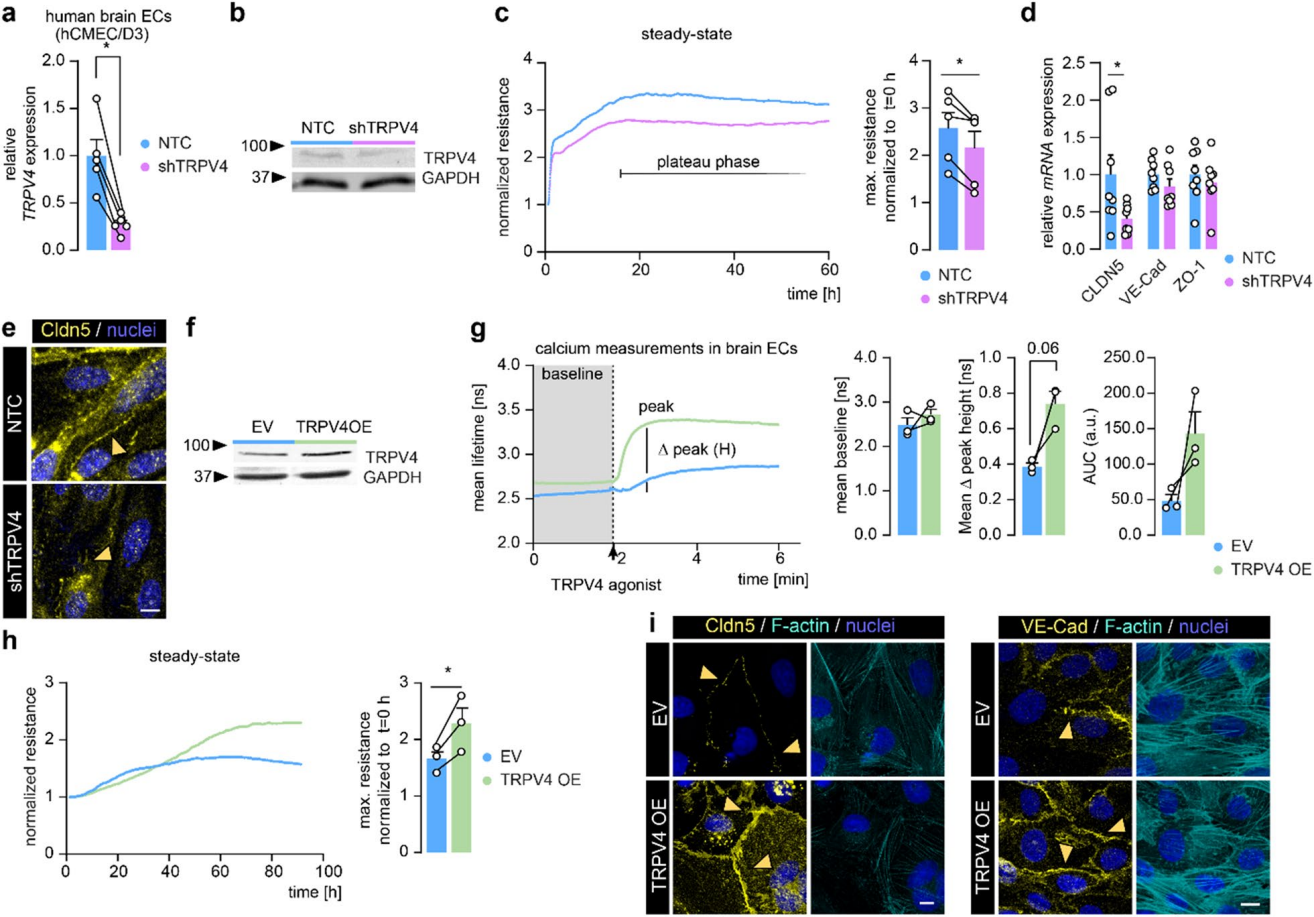
TRPV4 regulates barrier resistance and Cldn5 expression in human brain ECs. **a** *TRPV4* measured in knock down of TRPV4 (shTRPV4) compared to non-targeting shRNA control human brain ECs (NTC), N = 5. **b** Protein levels of TRPV4 in shTRPV4 cells compared to NTC cells. **c** TEER measurement of shTRPV4 and NTC brain ECs and quantification of max. resistance normalized to cell attachment (t = 0), N = 5. **d** mRNA expression of *CLDN5, VE-Cad* and *ZO-1* in shTRPV4 and NTC cells, N = 8. **e** Representative images of Cldn5 immunoreactivity in shTRPV4 cells and NTC cells, orange arrowheads indicate cell–cell junctions; scale bar: 25 µm. **f** Protein levels of TRPV4 in TRPV4 OE compared to EV cells. **g** Calcium baseline and TRPV4 agonist-mediated calcium response in TRPV4 OE compared to EV cells (GSK1016790A, 100 nM). Quantification of TRPV4 agonist-mediated calcium response, N = 3. **h** TEER measurement of TRPV4 OE and EV brain ECs and quantification of max. resistance normalized to t = 0, N = 3. **i** Representative images of Cldn5, VE-Cad, and F-actin immunoreactivity in TRPV4 OE and EV cells, orange arrowheads indicate cell–cell junctions; scale bar: 10 µm. Data is shown as mean ± SEM and an average of technical replicates in each biological replicate. Statistics were performed by paired Student’s t-test indicated by connecting lines (*p < 0.05)

To study the impact of altered endothelial TRPV4 levels on BBB function, we reduced TRPV4 levels (by mean 73%) in human brain ECs (hCMEC/D3) using a short hairpin knock down approach (shTRPV4) (Fig. 3a, b). We next quantified the maximum (max.) barrier resistance of shTRPV4 cells compared to non-targeting shRNA control cells (NTC) by TEER measurement. Although both cell types reached the plateau phase at a similar time, TRPV4 knock down resulted in a reduced barrier resistance compared to NTC (Fig. 3c). In line, expression of *CLDN5*, was reduced in shTRPV4 cells at the mRNA and protein level, whereas no significant differences were found for the mRNA of *zona occludens-1 (ZO-1)* and *VE-Cad* (Fig. 3d, e). Conversely, to investigate the consequences of increased TRPV4 expression on BBB function, we overexpressed TRPV4 in human brain ECs (TRPV4 OE). TRPV4 OE resulted in a two-fold increase in TRPV4 protein levels compared to empty vector (EV) control cells (Fig. 3f). Functionally, TRPV4 OE and EV cells displayed comparable intracellular calcium levels under resting conditions, but when stimulated by a selective TRPV4 agonist, TRPV4 OE cells evoked a higher calcium response compared to EV cells (Fig. 3g), indicating that the genetic overexpression lead to an elevation of functional TRPV4 channels. Furthermore, TRPV4 OE cells displayed an increased barrier resistance and elevated Cldn5 and VE-Cad levels (Fig. 3h, i). Together, TRPV4 expression levels impact brain EC barrier resistance under homeostatic conditions, potentially through its effect on the expression of Cldn5 and VE-Cad.

### Brain endothelial TRPV4 enhances BBB dysfunction under inflammatory conditions

**Fig. 4:**
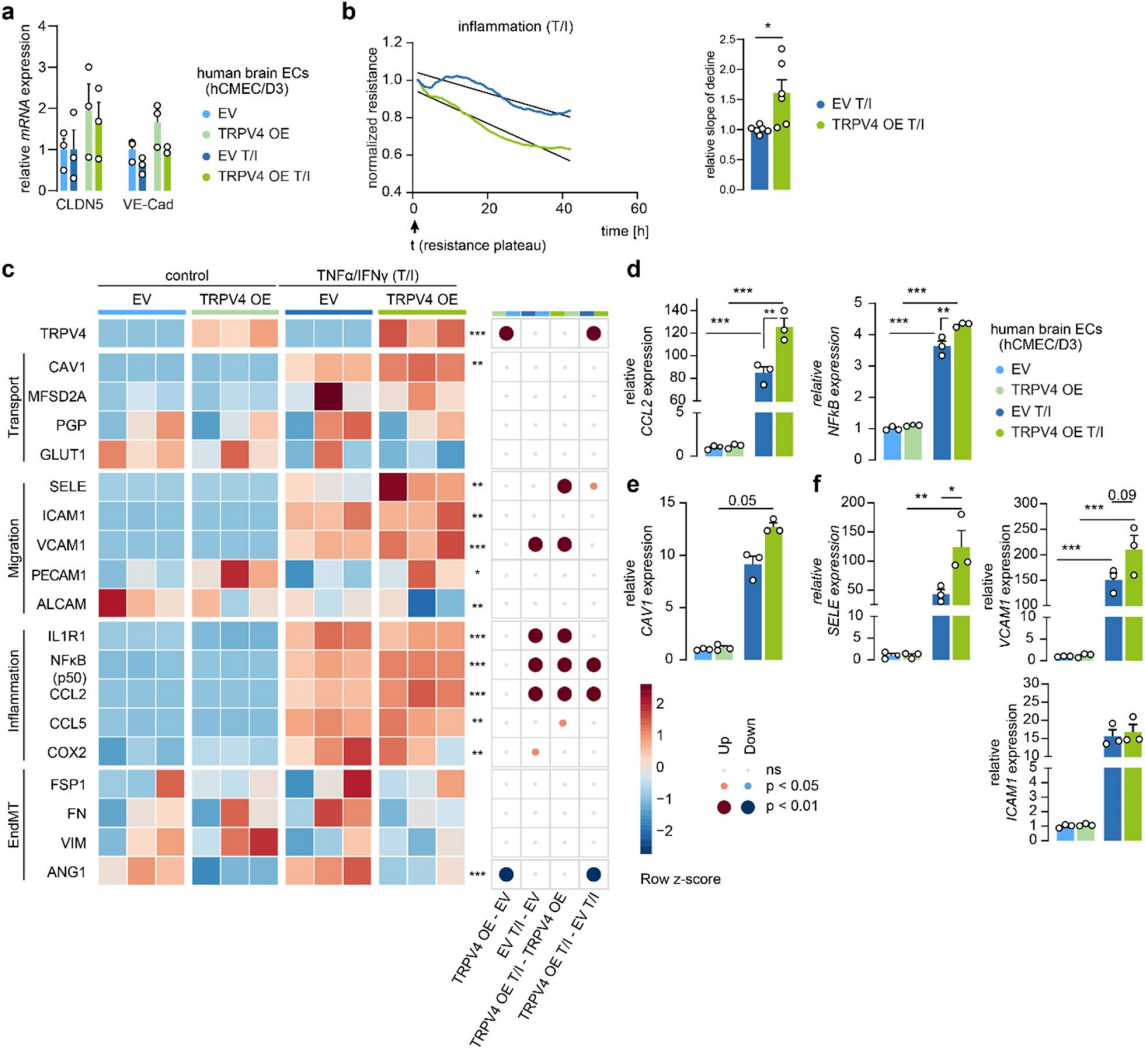
Enhanced TRPV4 expression in human brain ECs intensifies inflammatory phenotype during inflammatory insult. **a** *CLDN5* and *VE-Cad* in TRPV4 OE compared to EV cells under homeostatic (control) and inflammatory [TNFα/IFNγ (T/I)] conditions normalized to EV control. **b** TEER measurement of TRPV4 OE and EV brain ECs under inflammatory conditions normalized to resistance plateau and quantification of declining slope relative to EV cells, N = 6. **c** Heatmap visualizes gene expression profile of TRPV4 OE and EV brain ECs under homeostasis and inflammation. Accessed categories cover transporters, immune cell migration, inflammatory and EndMT markers, N = 3 **d**–**f** Examples of differentially expressed targets were re-plotted as bar graphs to visualize effect size. Data represents mean ± SEM. Comparison of four groups was performed using one-way ANOVA with Bonferroni multiple comparisons test or non-parametric Kruskal–Wallis test with Dunn’s test (*p < 0.05; **p < 0.01; ***p < 0.001)

Previous reports suggest that physiologically, without challenge, the activity of TRPV4 is largely suppressed, but inflammatory mediators can quickly evoke TRPV4-mediated responses [67]. Considering the inflammatory milieu in MS and the observed increase in endothelial TRPV4 levels, we explored phenotypic changes following TRPV4 OE in brain ECs in the presence and absence of inflammatory stimuli. The presence of TNFα/IFNγ (T/I), for 24 h did not significantly change the expression of *CLDN5* or *VE-Cad* both in EV and TRPV4 OE brain ECs (Fig. 4a). However, when measuring barrier properties of EV and TRPV4 OE brain ECs under these inflammatory conditions, we observed that the resistance of TRPV4OE cells declined at a higher rate compared to the EV cells, hinting towards accelerated impairment of the BBB (Fig. 4b). Further, we quantified in EV and TRPV4 OE cells, both homeostatic and T/I inflamed, the expression levels of genes implicated in BBB transport, immune cell migration, inflammatory responses, and endothelial to mesenchymal transition (EndMT), a process linked to BBB dysfunction [68], using multiplex qPCR (Fig. 4c). Inflammation markers like *CCL5* and *COX2* were generally increased upon inflammation, but in particular *NFκB* and *CCL2* were significantly induced in inflamed TRPV4 OE compared to inflamed EV cells (Fig. 4d). Expression levels of most transporters, including P-glycoprotein (*PGP)* and glucose transporter 1 (*GLUT1),* did not change, however, we observed a nearly tenfold increase in *Caveolin-1* (*CAV1*) levels upon inflammation in EV and 15 fold in TRPV4 OE cells (Fig. 4e). In line, we observed that inflammation evoked an increase in *E-selectin (SELE)*, *ICAM1* and *VCAM1*, which are key players in the multistep immune cell extravasation cascade. Specifically, *SELE* expression was potentiated in TRPV4 OE brain ECs and *VCAM1* showed a positive trend compared to EV cells under inflammation, while ICAM1 didn’t differ significantly (Fig. 4f). A few selected targets including *CAV1, ICAM1,* and *CCL2* were measured in shTRPV4 and NTC brain ECs under the same inflammatory conditions. Both *CAV1* and *ICAM1* were significantly less expressed by approximately tenfold in the inflamed shTRPV4 cells and *CCL2* showed a decreased trend compared to inflamed NTC cells (Additional file 1: Fig. S3a). Together, these findings suggest that elevated TRPV4 levels in inflamed brain ECs result in an accelerated decrease of barrier integrity and upregulation of genes related to inflammation and immune cell migration.

### Inhibiting TRPV4 activity reduces T cell diapedesis across the inflamed BBB

**Fig. 5:**
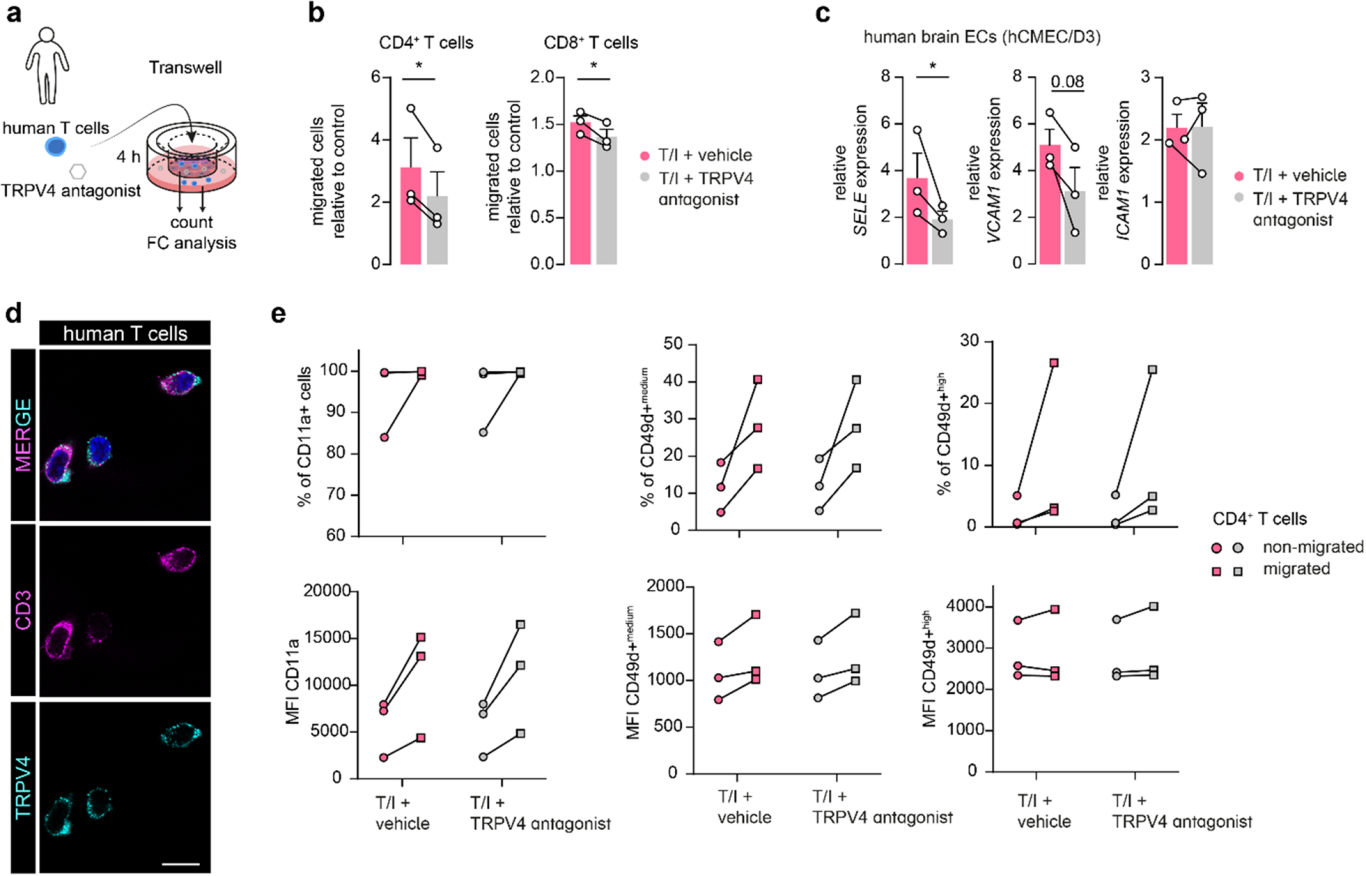
Inhibition of TRPV4 activity reduces T cell migration across the BBB in vitro. **a** Schematic of the static transwell set-up of 4 h T cell migration across human brain ECs treated with vehicle or TRPV4 antagonist. **b** Number of CD4^+^ and CD8^+^ T cells that migrated across stimulated (TNFα/IFNγ (T/I)) brain ECs treated with TRPV4 antagonist or vehicle normalized to unstimulated control, N experiments = 3, N CD4^+^ T cell donor = 2, N CD8^+^ T cell donor = 3. **c** *SELE, VCAM1,* and *ICAM1* expression in brain ECs treated equally to migration assay for 4 h, N = 3*.* **d** Representative image of CD3 and TRPV4 immunoreactivity in human T cells; scale bar: 10 µm. **e** Cell surface expression of CD11a and CD49d on migrated and non-migrated human CD4^+^ T cells quantified as an abundance of positive cells and median fluorescent intensity (MFI), N = 3. Data are shown as mean ± SEM and a comparison of two groups was performed using (ratio) paired Student’s t-test indicated by connecting lines (*p < 0.05)

Based on the findings above, we hypothesized that TRPV4 is involved in immune cell migration across the BBB, especially under inflammatory conditions. To this end, we quantified the total number of transmigrated T cells in the presence or absence of the selective TRPV4 inhibitor (GSK2193874, 1 µM). We used a static transwell-based system with a confluent monolayer of human brain ECs, which was subsequently stimulated with TNFα/IFNγ (T/I). Human CD4^+^ and CD8^+^ T cells were allowed to migrate across the brain EC monolayer for 4 h after which the total number of migrated T cells was assessed (Fig. 5a). Inhibition of TRPV4 resulted in a significant reduction of CD4^+^ and CD8^+^ T cells that migrated across brain EC monolayers compared to the vehicle condition (Fig. 5b). We further demonstrated that TRPV4 inhibition over 4 h caused reduced *SELE* expression in human brain ECs, while there was a trend towards reduced *VCAM1* expression whereas *ICAM1* expression was unaffected (Fig. 5c). However, as we observed TRPV4 expression in human T cells, in line with a previous report (Fig. 5d [69]), we wanted to rule out a direct effect of TRPV4 inhibition on the migratory profile of CD4^+^ T cells. As expected, the cell surface expression of CD11a and CD49d integrins, which are crucial for T cell migration across the BBB [70], as well as the percentage of CD49d^+^ (^medium and high^) cells, was higher in migrated compared to non-migrated T cells. However, expression levels were not influenced by TRPV4 inhibition, pointing towards an endothelial TRPV4-mediated effect during immune cell migration (Fig. 5e). Together, these findings suggest a direct role of TRPV4 activity during T cell migration over the inflamed human BBB, highlighting its potential as a druggable target to reduce T cell extravasation.

## Discussion

**Fig. 6:**
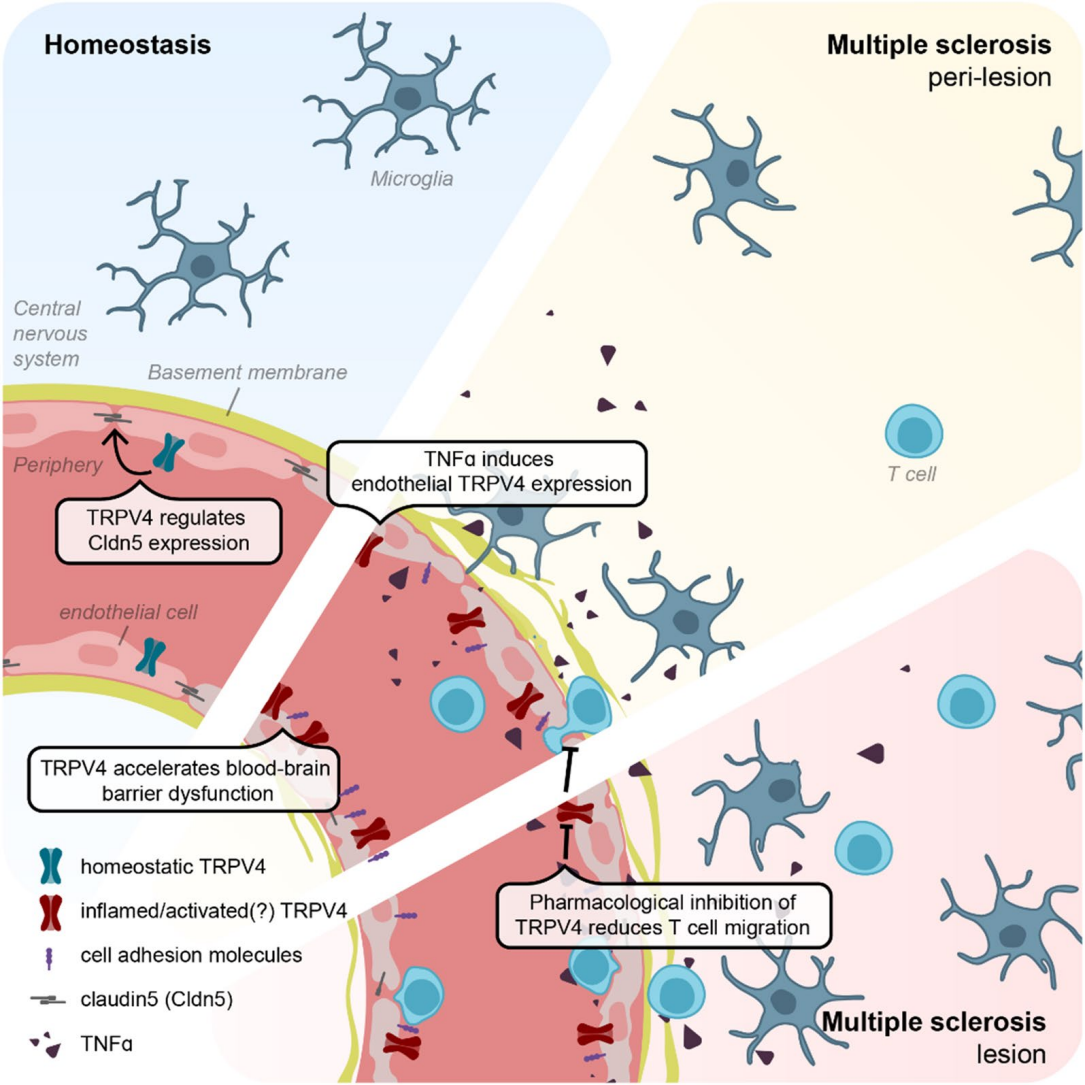
Schematic summary of the proposed mechanism underlying TRPV4 expression and function at the BBB in MS

BBB impairment and enhanced immune cell migration into the CNS parenchyma are at the core of MS lesion development and disease activity, but the directing molecular players of BBB dysfunction remain largely elusive. In our study, we provide the first indications for an altered expression and function of the ion channel TRPV4 in MS and consequent detrimental effects for the BBB. Specifically, we report an increased expression of TRPV4 in the endothelium of mixed A/I MS peri-lesion tissue compared to control brains. In the same tissue area, we found increased microglia-vessel proximity and we identified TNFα as a potential driver of enhanced endothelial TRPV4 expression. Our in vitro experiments further suggest different roles for endothelial TRPV4 in healthy and pathological states. Under resting conditions, TRPV4 levels contribute to BBB formation by regulating Cldn5 expression. Importantly, induced endothelial TRPV4 expression during inflammation accelerates BBB dysfunction and enhances inflammatory induction of key players of the immune cell migration cascade, suggesting TRPV4 as a regulator of endothelial inflammation. In line with this concept, inhibition of TRPV4 activity reduced CD4^+^ and CD8^+^ T cell migration across the inflamed brain endothelium and therefore presents a potential strategy to modulate BBB dysfunction and T cell extravasation in MS (Fig. 6).

TRPV4 is widely expressed in CNS cells including neurons, astrocytes, and microglia but has been extensively studied in its role in endothelial function and barrier homeostasis [41, 71, 72]. Here, we describe a region-specific elevated expression of TRPV4 in the brain endothelium of MS tissue. Specifically, TRPV4 levels are higher in the peri-lesional tissue of mixed A/I lesions compared to the lesion border and in the non-affected white matter of NNCs. Region-specific elevations of TRPV4, surrounding the site of pathology, have been reported in high-grade meningiomas, in a model of stroke (middle cerebral artery occlusion, (MCAO)) and experimental spinal cord injury (SCI), but these studies lack vascular specification [73–76]. Both in MCAO and SCI, TRPV4 expression peaked early at the site of injury after 18–24 h, indicative of an acute response [42]. Contrarily, the formation of MS lesions takes place over weeks to months, in which lesions undergo a non-linear evolution e.g. from active to inactive and potentially chronic state which is most prevalent in postmortem tissue [77]. The chronic inflammatory profile of the lesion could be one explanation for the moderate expression of TRPV4 in the lesion border of mixed A/I and CIA lesion tissue, as we observed it. Peri-lesional tissue, distal to the lesion in the same tissue block, presents notable changes including an increase in (activated) microglia potentially preceding lesion expansion [78, 79]. Hence, increased TRPV4 expression in this particular location of the MS lesion could confirm the role of TRPV4 in more acute inflammation. Further quantification of pre-active and active WM lesions will help to substantiate this concept, and add to previously reported alterations of ion channels in the BBB of MS patients [80]. Intriguingly, when studying TRPV4 in SCI, the authors propose TRPV4 localization at microglia-endothelial junctions, which matches our observations of pronounced endothelial TRPV4 expression upon the presence of directly adjacent microglia [42]. Furthermore, we found that microglia are closer to brain ECs in peri-lesional areas compared to control and lesion borders. Therefore, higher microglia-endothelial proximity and their shared local microenvironment represent a plausible mechanism for the increase of endothelial TRPV4 in peri-lesional MS tissue.

Microglia are central players in MS and undergo drastic morphological and functional changes during the disease course. Comparable to other neuroinflammatory diseases, microglia within MS lesions express higher activation markers and less P2RY12, a homeostatic marker, which also has been implied in vessel-microglia communication [81–84]. Activated microglia are one of the main producers of cytokines and chemokines in the CNS by which they can regulate and impair the nearby vasculature [85, 86]. We here describe that conditioned medium harvested from pro-inflammatory hiPSC-derived microglia triggered endothelial TRPV4 expression, at least partly via TNFα signaling. This upregulation was selective to TNFα within the tested cytokines including IFNγ, TGFβ, and Il1β. However, TNF inhibitor treatment could not fully reverse increased TRPV4 levels, suggesting that additional mediators in the medium could trigger TRPV4 expression. Importantly, microglia are not the only source of TNFα production. In mixed A/I lesions of MS tissue, we found both microglia and PVMs co-localizing with TNFα with a higher relative cell density of microglia over PVMs. In line with a previous report, TNFα reactivity was also present in the vasculature and significantly decreased in the lesion border compared to peri-lesion tissue [87]. It remains disputable why endothelial TRPV4 expression decreases in the lesion border, while TNFα is still present. An evident explanation could be the chronic active state of most of the investigated lesions, hence the acute response of the endothelium to TNFα subsided and as a consequence, basal TRPV4 expression is reinstalled. This acute to chronic shift upon prolonged TNFα exposure has been previously reported in peripheral endothelial cells, in which e.g. cell adhesion molecule expression declines after two days from the initial peak [88].

In our in vitro work, we modulate TRPV4 levels in brain ECs to understand the consequences of altered TRPV4 expression for the BBB. Interestingly, we found a context-dependent effect of TRPV4. First, under resting conditions, TRPV4 expression is linked to junction protein expression of Cldn5 and VE-Cad and, consistently, reducing TRPV4 impaired endothelial barrier resistance, while increasing TRPV4 expression increased barrier properties. In line, studies in keratinocytes indicate TRPV4’s importance for adherens junction formation via protein–protein interactions with β-catenin and E-cadherin at the N-terminus of TRPV4 [89]. Similarly, the cytoplasmic domain of TRPV4 in brain ECs could support tight and adherens junction formation by interaction with the actin cytoskeleton as shown for peripheral ECs [43]. The crucial role of TRPV4 in instating the endothelial barrier seems to be linked to its structure/location and not necessarily to its activity, as selective activation causes an increase in endothelial permeability [42]. Moreover, Rosenkranz et al*.*, showed that TRPV4 inhibition further increased murine BBB TEER which was an effect restricted to steady-state conditions [39]. Therefore, we hypothesize a stabilizing effect of TRPV4 for the BBB at baseline potentially based on its protein–protein interaction and low basal activity. Of note, this two-sided effect of TRPV4 on barrier function, by stabilizing intracellular junctions on one side and calcium-mediated barrier loss through i.e. reorganization of the actin cytoskeleton on the other, may also extend to peripheral tissues like lung endothelial/epithelial barriers and explain previous, seemingly contradictory, findings [30, 90, 91].

TNFα signaling in peripheral ECs is hypothesized to activate TRPV4 via autocrine signaling of adenosine triphosphate (ATP) [38, 86]. Further, TRPV4 channels respond to inflammatory cytokines and pro-inflammatory lipid derivatives, which are abundantly present during neuroinflammation and MS [92, 93]. Matching, activation of TRPV4 channels has been reported in other neurological diseases like AD and cerebral ischemia leading to cell death, inflammatory cytokine release, and reactive oxygen species production [72, 94]. Our study explores the consequences of endothelial TRPV4 overexpression to estimate the effect of higher TRPV4 levels in peri-lesional MS vasculature. The lentiviral-induced overexpression results in similar TRPV4 levels as observed in brain endothelial cells treated with TNFα. Under inflammation, we observed upregulation of NFκB (p50) and CCL2 in TRPV4 OE cells. A recent study in mice with pilocarpine-induced status epilepticus highlights the connection between TRPV4 activation and NFκB nuclear translocation in neurons [44]. Reports in mice and human peripheral ECs confirm that TRPV4 activation drives the endothelium to a pro-inflammatory signature and channel inhibition reduces in particular candidates of the NFκB pathway including cell adhesion molecules and pro-inflammatory cytokine release [43, 44, 95, 96]. Consequently, TRPV4 signaling could further perpetuate local endothelial inflammation. We also observed a steeper decline of barrier resistance in inflamed endothelial cells with heightened TRPV4 expression compared to control cells, suggesting an accelerated BBB dysfunction through TRPV4. In line, inflamed TRPV4 OE cells express enhanced Cav1, a marker for impaired BBB function. Contextualizing these changes into MS, high endothelial TRPV4 activity promotes a shift in brain ECs towards a pro-inflammatory state, which quickens BBB activation and integrity loss, thus favoring immune cell migration. Consequently, acutely inhibiting TRPV4 has been shown to be beneficial in multiple CNS-disease models like cuprizone-induced demyelination and SCI [40, 42, 97]. However, Rosenkranz et al*.* specifically compared TRPV4^−/−^ mice and littermates in experimental autoimmune encephalomyelitis (EAE), a well-known animal model for MS, and did not observe apparent difference in clinical score or BBB leakage [39]. Importantly, as TRPV4 deficiency was induced by a constitutive knockout in that specific study, BBB alterations or compensation mechanisms during development could be present thereby masking/counteracting a potential beneficial effect of TRPV4 activity deficiency on the disease outcome. Inhibiting TRPV4 activity acutely during the EAE experiment could delineate its potential as a druggable target, without developmental influences. Importantly, the systematic application of TRPV4 inhibitors may impact other peripheral tissues expressing TRPV4 such as the lung and pancreas [91, 98]. To monitor potential adverse effects it is crucial to perform a comprehensive assessment of other organs functions during the EAE experiment.

As one of the hallmarks of MS pathology, T cell extravasation, is a pivotal event for lesion development. Here, we provide evidence for the relevance of TRPV4 activity during T cell migration. Firstly, TRPV4 mediates E-selectin (SELE) expression, an important player of the immune cell extravasation cascade across the BBB [99]. While inflamed TRPV4 OE cells showed higher expression of E-selectin, TRPV4 inhibition during inflammation decreased its expression. Secondly, we observed that total migration of both CD4^+^ and CD8^+^ human T cells was reduced upon TRPV4 inhibition compared to vehicle-treated inflamed brain ECs. The blockage of TRPV4 occurred acutely, which suggests a direct role of TRPV4-mediated calcium signaling during the T cell migration cascade. Calcium-driven cytoskeleton remodeling is essential for leukocyte transmigration and TRPV4 is critically involved both in tubulin- and actin-dependent cell movement [61, 100, 101]. Similarly, another member of the TRP channel family, TRPC6, has been implicated in leukocyte transendothelial migration, distinctly during the diapedesis step [102]. Together, our data provide an initial incentive to study TRPV4 inhibition to limit immune cell migration across the BBB.

## Conclusion

In summary, we present evidence for a novel role for TRPV4 at the human BBB and in MS. Peri-lesional areas of MS brain lesions show increased TRPV4 expression as well as enhanced microglia-endothelial proximity. In vitro, TRPV4 expression modulates barrier formation by regulating Cldn5 expression under physiological conditions, while heightened TRPV4 during inflammation is associated with an increase of pro-inflammatory markers and accelerated BBB impairment. Acutely inhibiting TRPV4 reduces T cell migration across the BBB which opens up an avenue towards reinstating BBB function in MS.

### Supplementary Information

**Additional file 1:** **Figure S1.** Ubiquitous TRPV4 expression in the human brain. **Figure S2.** iPSC microglia phenotyping and TRPV4 in HLA-DR + cells. **Figure S3.** Reduced TRPV4 expression lowers inflammatory profile in brain ECs during inflammation. **Figure S4.** Flow cytometry gating strategy of human T cells

**Additional file 2: Figure S1.** Original Western blot images. **a** Immunoblot of TRPV4 from brain ECs treated with TNFα (left) or transduced with the TRPV4 knock down construct (shTRPV4) or control (NTC) (right). **b** Immunoblot of GAPDH serving as a reference protein  for samples used in **a**. **c** Immunoblot of TRPV4 and GAPDH from brain ECs transduced with the TRPV4 overexpression construct (TRPV4OE) or control (EV).

## Data Availability

All data presented in this study are available from the corresponding author upon reasonable request.

## Abbreviations

A/I: Mixed active/inactive
AJs: Adherens junctions
ATP: Adenosine triphosphate
AUC: Area under the curve
BBB: Blood–brain barrier
Brain ECs: Brain microvascular endothelial cell
Cav1: Caveolin-1
CIA: Chronic inactive
Cldn5: Claudin-5
CNS: Central nervous system
Coll IV: Collagen IV
DMSO: Dimethyl sulfoxide
EAE: Experimental autoimmune encephalomyelitis
EndMT: Endothelial to mesenchymal transition
EV: Empty vector
FBS: Fetal bovine serum
FCS: Fetal calf serum
FLIM: Fluorescent lifetime imaging
Glut1: Glucose transporter 1
hCMEC/D3: Human cerebral microvascular endothelial cell line
hiPSC: Human induced pluripotent stem cells
HLA-DR: Major Histocompatibility Complex, Class II, DR
ICAM1: Intercellular adhesion molecule-1
IFNγ: Interferon gamma
IL1β: Interleukin-1 beta
LPS: Lipopolysaccharide
MCAO: Middle Cerebral Artery Occlusion
M-CSF: Macrophage colony-stimulating factor
MG: Microglia
MS: Multiple sclerosis
NFκB: Nuclear factor kappa-light-chain-enhancer of activated B cells
NNC: Non-neurological controls
NTC: Non-targeting control
P/S: Penicillin–streptomycin
Pgp: P-glycoprotein
PLP: Proteolipid Protein
pMBMECs: Primary mouse brain microvascular endothelial cells
PVM: Perivascular macrophages
qRT-PCR: Quantitative real-time PCR
RPMI: Roswell Park Memorial Institute
SCI: Experimental spinal cord injury
Sele: E-selectin
ShRNA: Short hairpin RNA
T/I: TNFα / IFNγ
TEER: Trans endothelial electric resistance
TGFβ: Transforming growth factor-β
TJs: Tight junctions
TNFα: Tumor necrosis factor-α
TRP: Transient receptor potential
TRPV4: Transient Receptor Potential Cation Channel Subfamily V Member 4
TRPV4 OE: TPRV4 overexpressing cells
UEA-I: Ulex Europaeus Agglutinin I
VE-Cad: VE-cadherin
WM: White matter
ZO-1: Zona occludens-1
