# Supplementary material for: Inflammation-induced TRPV4 channels exacerbate blood–brain barrier dysfunction in multiple sclerosis
Source: J Neuroinflammation. 2024 Mar 23;21:72. doi: 10.1186/s12974-024-03069-9 (PMC10960997; doi:10.1186/s12974-024-03069-9)
Supplement: Supplementary file 1 — Additional file 1: Figure S1. Ubiquitous TRPV4 expression in the human brain. Figure S2. iPSC microglia phenotyping and TRPV4 in HLA-DR + cells. Figure S3. Reduced TRPV4 expression lowers inflammatory profile in brain ECs during inflammation. Figure S4. Flow cytometry gating strategy of human T cells [file 12974_2024_3069_MOESM1_ESM.docx]

**Supplementary Information – Additional file 1**

**Inflammation-induced TRPV4 channels exacerbate blood-brain barrier dysfunction in multiple sclerosis**

Cathrin E. Hansen^1,2,3^*, Alwin Kamermans^1,2,3^, Kevin Mol^4^, Kristina Berve^6^, Carla Rodriguez-Mogeda^1,2,3^, Wing Ka Fung^1^, Bert van het Hof^1^, Ruud Fontijn^1^, Susanne M.A. van der Pol^1^, Laura Michalick^7,8^, Wolfgang M. Kuebler^7-10^, Boyd Kenkhuis^11,14^, Willeke van Roon-Mom^11^, Wolfgang Liedtke^12,13^ Britta Engelhardt^6^, Gijs Kooij^1,2,3,5^, Maarten E. Witte^1,2,3,5^ and Helga E. de Vries^1,2,3^*

^1^Amsterdam UMC location Vrije Universiteit Amsterdam, Department of Molecular Cell Biology and Immunology, De Boelelaan 1117, Amsterdam, The Netherlands

^2^Amsterdam Neuroscience, Amsterdam UMC, Amsterdam, The Netherlands

^3^MS Center Amsterdam, Amsterdam UMC Location VU Medical Center, Amsterdam, The Netherlands

^4^Amsterdam UMC location University of Amsterdam, Department of Biomedical Engineering and Physics, Meibergdreef 9, Amsterdam, the Netherlands

^5^Amsterdam institute for Infection and Immunity, Amsterdam UMC, Amsterdam, The Netherlands

^6^Theodor Kocher Institute, University of Bern, Bern, Switzerland

^7^Institute of Physiology, Charité-Universitätsmedizin Berlin, corporate member of the Freie Universität Berlin and Humboldt Universität to Berlin, Berlin, Germany

^8^DZHK (German Centre for Cardiovascular Research), partner site Berlin, Germany

^9^Keenan Research Centre for Biomedical Science, St. Michael’s Hospital, Toronto, Ontario, Canada

^10^Departments of Surgery and Physiology, University of Toronto, Toronto, Ontario, Canada

^11^Department of Human Genetics, Leiden University Medical Center Leiden, Leiden, The Netherlands

^12^Department of Neurology, Duke University, Durham, NC, USA

^13^Department of Molecular Pathobiology, New York University College of Dentistry, NC, USA

^14^UK Dementia Research Institute at University of Edinburgh, Edinburgh, UK

* Corresponding authors: C.E.H. (email: [c.e.hansen@amsterdamumc.nl](mailto:c.e.hansen@amsterdamumc.nl), tel: +31 (0) 204448080) and H.E.d.V. (email: [he.devries@amsterdamumc.nl](mailto:he.devries@amsterdamumc.nl), tel: +31 (0) 204448080)

**
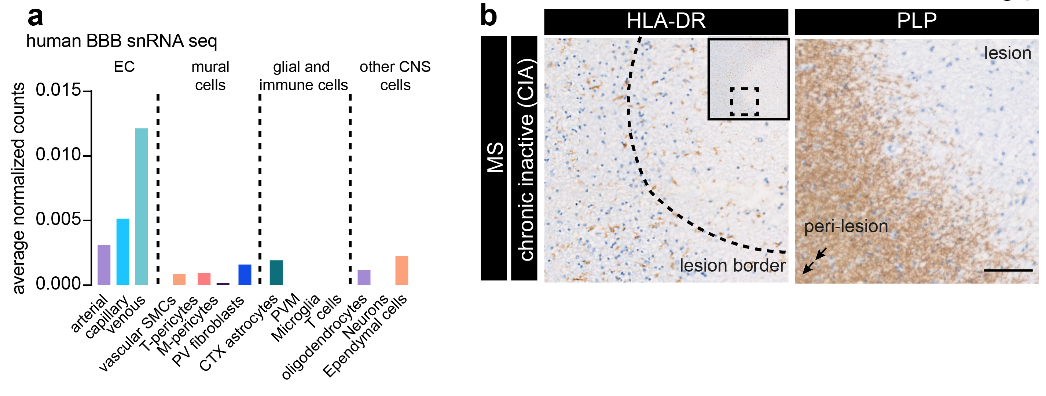
**

**Fig. SI1** **Ubiquitous TRPV4 expression in the human brain**

**a** snRNA sequencing data showing TRPV4 expression in different CNS cells; obtained from the human brain vascular atlas (Yang et al., 2022, (68)). **b** Representative images of HLA-DR and PLP immunoreactivity in CIA WM lesion tissue. Main image shows a magnification of the lesion border (dotted line), arrows indicate peri-lesional tissue distal from the lesion border. Solid squares present lesion overview and dotted square the panel; scale bar: 5 mm (insert), 400 µm (magnification).

**
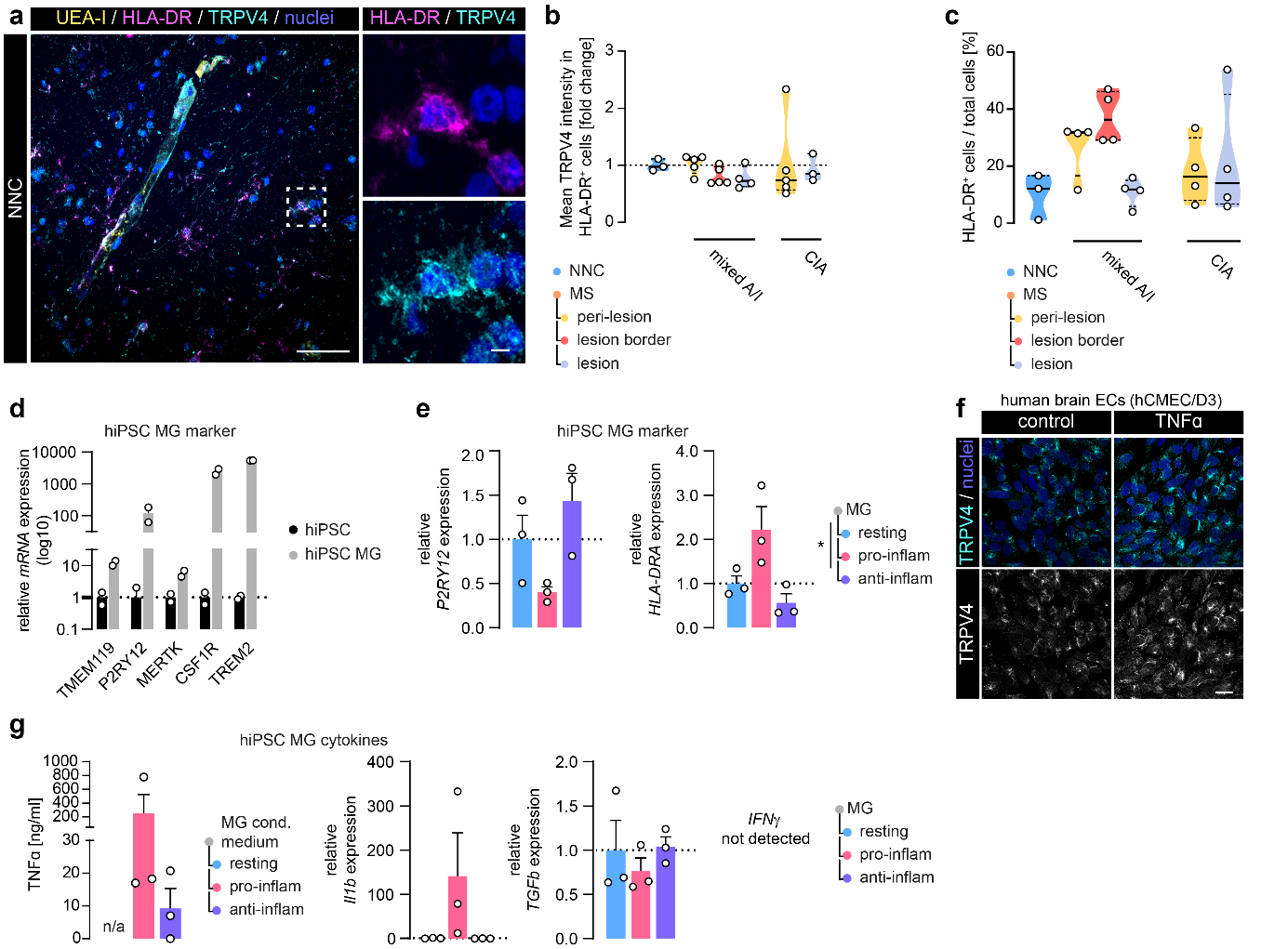
**

**Fig. SI2** **iPSC microglia phenotyping and TRPV4 in HLA-DR^+^ cells**

**a** Representative confocal image of UEA-I, HLA-DR and TRPV4 immunoreactivity in NNC; scale bar: 50 µm, panel 5 µm. **b** Quantification of TRPV4 levels in HLA-DR^+^ cells comparing NNC, peri-lesion, lesion border and lesion. **c** Quantification of HLA-DR^+^ cells from total cell count (percentage) in mixed A/I and CIA lesions compared to NNC; NNC: N cases=3, MS tissue (mixed A/I: N cases=3, N lesions=5; CIA: N cases=4, N lesions=5). Violin plots show median ± quartiles. **d** mRNA levels of microglia marker (TMEM119, P2RY12, MERTK, CSF1R, TREM2) in hiPSCs and hiPSC microglia (MG), N=2. **e** mRNA levels of homeostatic microglia marker P2RY12 and activation marker HLA-DRA in hiPSC MG with different phenotypes (resting, pro-inflam., anti-inflam.), N=3. **f** Representative images of TRPV4 immunoreactivity in human brain ECs (hCMEC/D3) untreated (control) and treated with TNFα for 24 h, scale bar: 25 µm. **g**Protein and mRNA levels of cytokines (TNFα, Il1β and TGFβ) in hiPSC MG, N=3. Data are shown as mean ± SEM and statistics were calculated by one-way ANOVA and comparison to resting condition using Dunnetts test (*p<0.05).

**
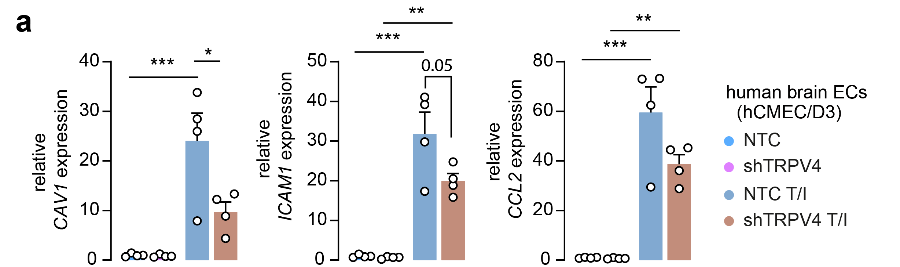
**

**Fig. SI3** **Reduced TRPV4 expression lowers inflammatory profile in brain ECs during inflammation**

**a** mRNA levels of CAV1, ICAM1 and CCL2 in shTRPV4 and NTC brain ECs under homeostatic and inflammatory (T/I) conditions, N=4. Data represents mean ± SEM and an average of technical triplicates in each biological replicate. Comparison of four groups was performed using one-way ANOVA with Bonferroni multiple comparisons test or non-parametric Kruskal-Wallis test with Dunn’s test (*p<0.05; **p<0.01, ***p<0.001).

**
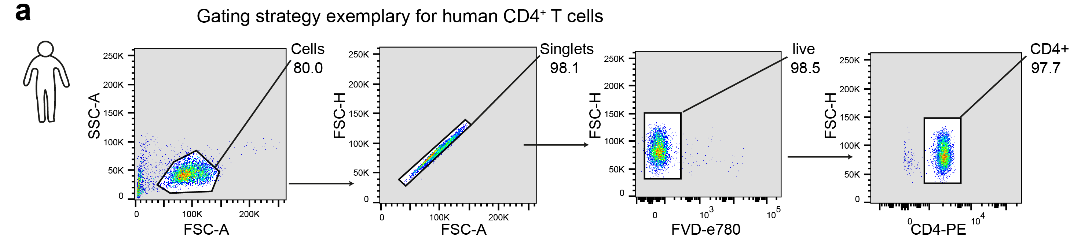
**

**Fig. S4** **Flow cytometry gating strategy of human T cells**

**a** Gating strategy for human T cells: After selection of cells based on the FSC-A and SCC-A and selection of singlets based on the FSC-A vs. FSC-H, dead cells were excluded by FVD eFluor780 positivity. Finally, CD4^+^ T cells were selected.
