# Supplementary material for: Inflammation-induced TRPV4 channels exacerbate blood–brain barrier dysfunction in multiple sclerosis
Source: J Neuroinflammation. 2024 Mar 23;21:72. doi: 10.1186/s12974-024-03069-9 (PMC10960997; doi:10.1186/s12974-024-03069-9)
Supplement: Supplementary file 2 — Additional file 2: Figure S1. Original Western blot images. a Immunoblot of TRPV4 from brain ECs treated with TNFα (left) or transduced with the TRPV4 knock down construct (shTRPV4) or control (NTC) (right). b Immunoblot of GAPDH serving as a reference protein for samples used in a. c Immunoblot of TRPV4 and GAPDH from brain ECs transduced with the TRPV4 overexpression construct (TRPV4OE) or control (EV). [file 12974_2024_3069_MOESM2_ESM.docx]

**Supplementary Information – Additional file 2**

**Inflammation-induced TRPV4 channels exacerbate blood-brain barrier dysfunction in multiple sclerosis**

Cathrin E. Hansen^1,2,3^*, Alwin Kamermans^1,2,3^, Kevin Mol^4^, Kristina Berve^6^, Carla Rodriguez-Mogeda^1,2,3^, Wing Ka Fung^1^, Bert van het Hof^1^, Ruud Fontijn^1^, Susanne M.A. van der Pol^1^, Laura Michalick^7,8^, Wolfgang M. Kuebler^7-10^, Boyd Kenkhuis^11,14^, Willeke van Roon-Mom^11^, Wolfgang Liedtke^12,13^ Britta Engelhardt^6^, Gijs Kooij^1,2,3,5^, Maarten E. Witte^1,2,3,5^ and Helga E. de Vries^1,2,3^*

^1^Amsterdam UMC location Vrije Universiteit Amsterdam, Department of Molecular Cell Biology and Immunology, De Boelelaan 1117, Amsterdam, The Netherlands

^2^Amsterdam Neuroscience, Amsterdam UMC, Amsterdam, The Netherlands

^3^MS Center Amsterdam, Amsterdam UMC Location VU Medical Center, Amsterdam, The Netherlands

^4^Amsterdam UMC location University of Amsterdam, Department of Biomedical Engineering and Physics, Meibergdreef 9, Amsterdam, the Netherlands

^5^Amsterdam institute for Infection and Immunity, Amsterdam UMC, Amsterdam, The Netherlands

^6^Theodor Kocher Institute, University of Bern, Bern, Switzerland

^7^Institute of Physiology, Charité-Universitätsmedizin Berlin, corporate member of the Freie Universität Berlin and Humboldt Universität to Berlin, Berlin, Germany

^8^DZHK (German Centre for Cardiovascular Research), partner site Berlin, Germany

^9^Keenan Research Centre for Biomedical Science, St. Michael’s Hospital, Toronto, Ontario, Canada

^10^Departments of Surgery and Physiology, University of Toronto, Toronto, Ontario, Canada

^11^Department of Human Genetics, Leiden University Medical Center Leiden, Leiden, The Netherlands

^12^Department of Neurology, Duke University, Durham, NC, USA

^13^Department of Molecular Pathobiology, New York University College of Dentistry, NC, USA

^14^UK Dementia Research Institute at University of Edinburgh, Edinburgh, UK

* Corresponding authors: C.E.H. (email: [c.e.hansen@amsterdamumc.nl](mailto:c.e.hansen@amsterdamumc.nl), tel: +31 (0) 204448080) and H.E.d.V. (email: [he.devries@amsterdamumc.nl](mailto:he.devries@amsterdamumc.nl), tel: +31 (0) 204448080)


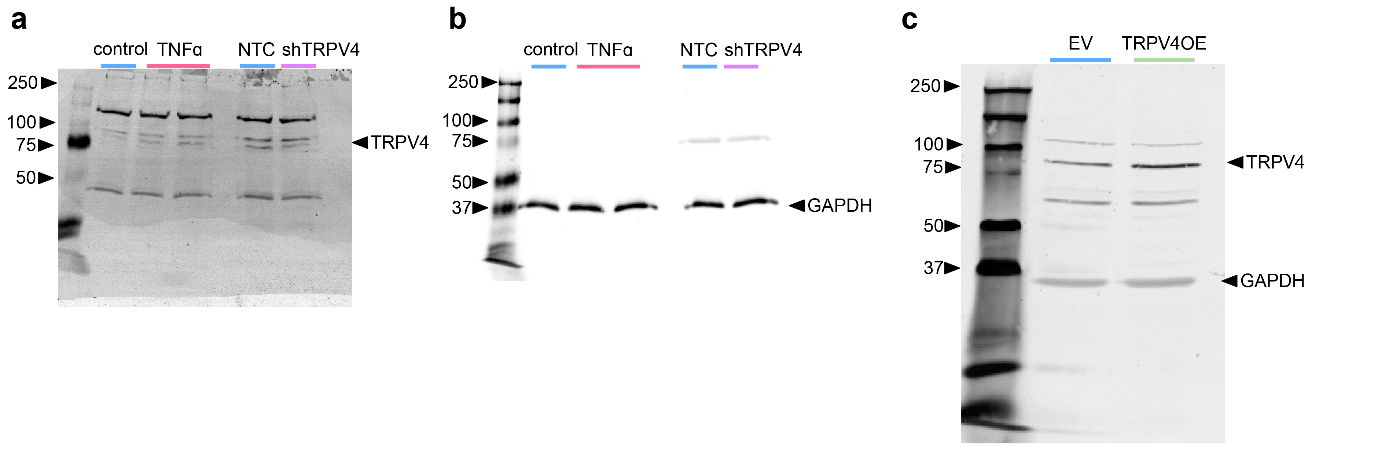


**Fig. S1 Original Western blot images a** Immunoblot of TRPV4 from brain ECs treated with TNFα (left) or transduced with the TRPV4 knockdown construct (shTRPV4) or control (NTC) (right). **b** Immunoblot of GAPDH serving as a reference gene for samples used in a. **c** Immunoblot of TRPV4 and GAPDH from brain ECs transduced with the TRPV overexpression construct (TRPV4OE) or control (EV).
